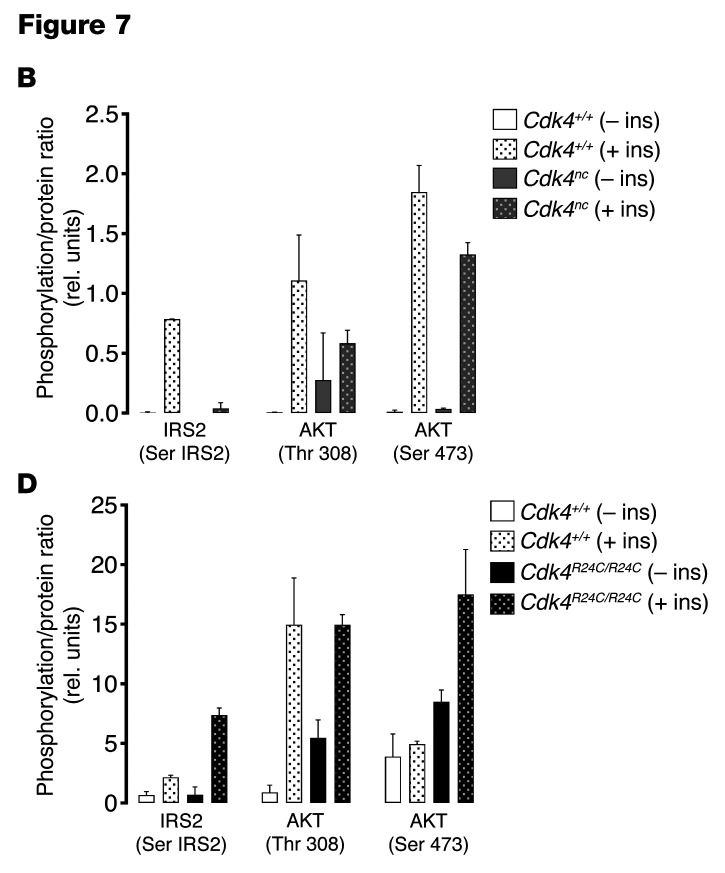# CDK4 is an essential insulin effector in adipocytes

**DOI:** 10.1172/JCI162359

**Published:** 2022-07-01

**Authors:** Sylviane Lagarrigue, Isabel C. Lopez-Mejia, Pierre-Damien Denechaud, Xavier Escoté, Judit Castillo-Armengol, Veronica Jimenez, Carine Chavey, Albert Giralt, Qiuwen Lai, Lianjun Zhang, Laia Martinez-Carreres, Brigitte Delacuisine, Jean-Sébastien Annicotte, Emilie Blanchet, Sébastien Huré, Anna Abella, Francisco J. Tinahones, Joan Vendrell, Pierre Dubus, Fatima Bosch, C. Ronald Kahn, Lluis Fajas

Original citation: *J Clin Invest*. 2016;126(1):335–348. https://doi.org/10.1172/JCI81480

Citation for this corrigendum: *J Clin Invest*. 2022;132(13):e162359. https://doi.org/10.1172/JCI162359

Following the publication of this article, the authors realized that errors were made during manuscript preparation. Figure 3J and Figure 3L, illustrating the insulin sensitivity test in the *Cdk4^nc^* and *Cdk^R24C/R24C^* mice, respectively, were not representative. Specifically, independent experiments using mice with different ages were aggregated in the results. The correct Figure 3J now shows insulin tolerance tests (ITTs) in 24-week-old *Cdk4^+/+^* and *Cdk4^nc^* (*n* = 5), and the correct Figure 3L shows ITT in 30-week-old *Cdk^R24C/R24C^* mice (*n* = 5–11). In contrast with the original published data, the corrected data do not show statistical significance in the insulin sensitivity tests.

In addition, the quantification in Figure 7B did not correspond with the immunoblot image in Figure 7A. Therefore, new quantification is now shown in Figure 7B. Data from one control mouse (lane 5) blot indicates no response to insulin, as assessed by the absence of IRS and AKT phosphorylation, which signifies failure of insulin injection. Consequently, this was removed from the quantification shown in Figure 7B. Due to the sample size limitation, statistical analyses in Figure 7, B and D, were removed.

For clarity, the authors have added information to the figure legends about the ages of mice for additional experiments shown in Figures 1, 3, 4, 5, and 7 and Supplemental Figures 1, 2, and 5.

The *Journal* has published an online version of the original article with the unreliable statements crossed out and the modified text highlighted in red (Supplemental File, Redaction). The authors have confirmed the accuracy of the data and that the corrected paper is reliable.

The authors regret the errors and the possible confusion generated to the readers of the *Journal*.

## Supplementary Material

Supplemental data

## Figures and Tables

**Figure:**
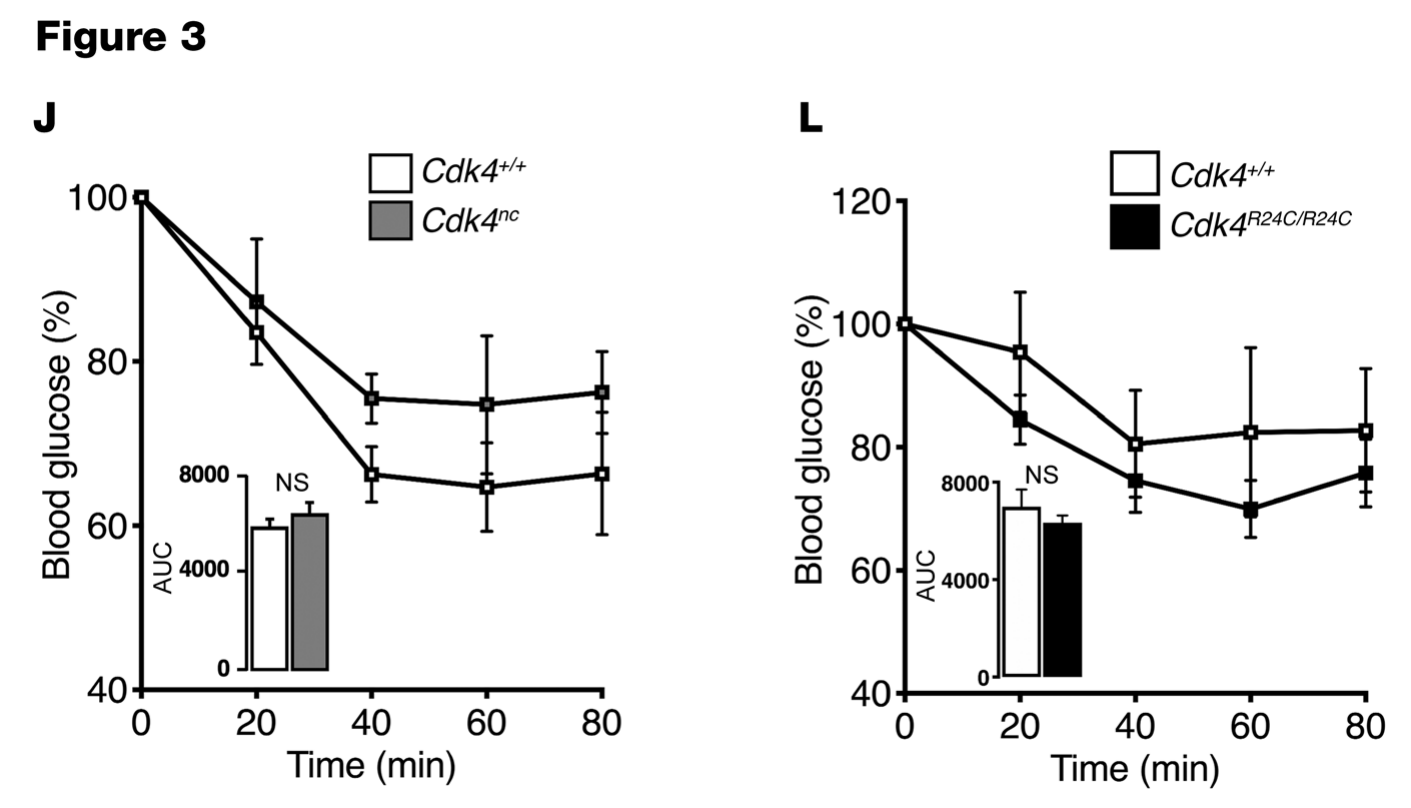


**Figure:**