# Supplementary material for: CDK4 is an essential insulin effector in adipocytes
Source: J Clin Invest. 2022 Jul 1;132(13):e162359. doi: 10.1172/JCI162359 (PMC9246374; doi:10.1172/JCI162359)
Supplement: Supplemental data [file jci-132-162359-s157.pdf]

CDK4 is an essential insulin effector in adipocytes

Sylviane Lagarrigue,[1,2] Isabel C. Lopez-Mejia,[1] Pierre-Damien Denechaud,[1] Xavier Escoté,[1] Judit Castillo-Armengol,[1] Veronica Jimenez,[3] Carine Chavey,[2] Albert Giralt,[1] Qiuwen Lai,[1] Lianjun Zhang,[4] Laia Martinez-Carreres,[1] Brigitte Delacuisine,[1] Jean-Sébastien Annicotte,[2,5] Emilie Blanchet,[2] Sébastien Huré,[1,2] Anna Abella,[6] Francisco J. Tinahones,[7,8] Joan Vendrell,[9] Pierre Dubus,[10] Fatima Bosch,[3] C. Ronald Kahn,[11] and Lluis Fajas[1,2]

[1]Department of Physiology, Université de Lausanne, Lausanne, Switzerland. [2]Institut de Génétique Moléculaire de Montpellier (IGMM), Université de Montpellier, Montpellier, France. [3]Center of Animal Biotechnology and Gene Therapy and Department of Biochemistry and Molecular Biology, School of Veterinary Medicine, Universitat Autònoma de Barcelona, Bellaterra, and Centro de Investigación Biomédica en Red de Diabetes y Enfermedades Metabólicas Asociadas, Barcelona, Spain. [4]Translational Tumor Immunology, Ludwig Center for Cancer Research, Université de Lausanne, Epalinges, Switzerland. [5]European Genomic Institute for Diabetes, Université Lille Nord de France, UMR 8199 CNRS, Lille, France. [6]INSERM U834, Montpellier, France. [7]Unidad de Gestión Clínica de Endocrinología y Nutrición, Hospital Universitario Virgen de la Victoria, Málaga, Spain. [8]Centro de Investigación Biomédica en Red-Fisiopatología de la Obesidad y la Nutrición (CIBERobn CB06/003), Instituto de Salud Carlos III, Madrid, Spain. [9]CIBERDEM, Institut d'Investigació Pere Virgili, Universitat Rovira i Virgili, Hospital Universitari Joan XXIII, Tarragona, Spain. [10]EA2406, Histologie et pathologie moléculaire des tumeurs, Université de Bordeaux, Bordeaux, France. [11]Section on Integrative Physiology and Metabolism, Joslin Diabetes Center, Harvard Medical School, Boston, Massachusetts, USA.

Insulin resistance is a fundamental pathogenic factor that characterizes various metabolic disorders, including obesity and type 2 diabetes. Adipose tissue contributes to the development of obesity-related insulin resistance through increased release of fatty acids, altered adipokine secretion, and/or macrophage infiltration and cytokine release. Here, we aimed to analyze the participation of the cyclin-dependent kinase 4 (CDK4) in adipose tissue biology. We determined that white adipose tissue (WAT) from CDK4-deficient mice exhibits impaired lipogenesis and increased lipolysis. Conversely, lipolysis was decreased and lipogenesis was increased in mice expressing a mutant hyperactive form of CDK4 (CDK4^{R24C}). ~~A global kinome analysis of CDK4-deficient mice following insulin stimulation revealed that insulin signaling is impaired in these animals. We determined that insulin activates the CCND3-CDK4 complex, which in turn phosphorylates insulin receptor substrate 2 (IRS2) at serine 388, thereby creating a positive feedback loop that maintains adipocyte insulin signaling.~~ We performed a global kinome analysis and found that mice lacking Cdk4 had impaired insulin signaling in the adipose tissue. Interestingly, our results demonstrated that insulin activates the cyclin D3-CDK4 complex, which, in turn, phosphorylates the insulin receptor substrate 2 (IRS2) at the Ser 388, likely creating a positive feedback loop to maintain adipocyte insulin signaling. Furthermore, we found that CCND3 expression and IRS2 serine 388 phosphorylation are increased in human obese subjects. Together, our results demonstrate that CDK4 is a major regulator of insulin signaling in WAT.

Introduction

Insulin signaling is a versatile system that coordinates growth, proliferation, and development of multiple tissues and organs by controlling metabolic processes that accommodate the energy needs of cellular function (1). Defects in insulin signaling contribute to insulin resistance, a common complication of obesity that occurs early in the pathogenesis of type 2 diabetes and cardiovascular disease (2, 3). Insulin response depends on tissue and cellular functions. In white adipose tissue (WAT), insulin signaling regulates lipid synthesis (1) and glucose transport (4–6) and represses lipolysis (7). However, the exact mechanism by which insulin signaling coordinates regulated cellular functions is not fully understood. Cyclin-dependent kinase 4 (CDK4) plays an important role in the G_1/S transition of the cell cycle. Its kinase activity is regulated through interaction with the D-type cyclins (CCND1, CCND2, and CCND3) (8). The resulting cyclin D–CDK4 complexes catalyze the phosphorylation of the members of the retinoblastoma (RB) protein family (RB1, RBL1, and RBL2). Phosphorylation of RB1 by cyclin D–CDK4 releases the E2F transcription factors, thereby ensuring the expression of genes required for cell-cycle progression (9). Conversely, members of the family of CDK inhibitors (INK and CIP/KIP) block CDK activity in response to quiescence stimuli. Many studies have assessed the roles of CDK4 in cell growth, proliferation, and cancer (10), but the role of CDK4 in adipose tissue function has never been explored. The most marked phenotypes of mice lacking CDK4 (*Cdk4*$^{neo/neo}$) are reduced body size and insulin-deficient diabetes due to a severe decrease in pancreatic β cell growth (11). β Cell–specific reexpression of the *Cdk4*^{R24C} allele renders CDK4 resistant to the inhibitory effects of INK4 proteins (12) and restores β cell proliferation and normoglycemic conditions (13). Interestingly, CDK4 reexpression in pancreatic β cells does not rescue body size reduction, suggesting that this phenotype is not due to endocrine defects secondary to decreased insulin levels. We previously demonstrated that CDK4 regulates adipogenesis, suggesting a role of CDK4 in WAT function (14).

Authorship note: S. Lagarrigue and I.C. Lopez-Mejia contributed equally to this work.
Conflict of interest: The authors have declared that no conflict of interest exists.
Submitted: February 12, 2015; **Accepted:** November 6, 2015.
Reference information: *J Clin Invest*. 2016;126(1):335–348. doi:10.1172/JCI81480.

Results

CDK4 activity is positively correlated with WAT mass. The first

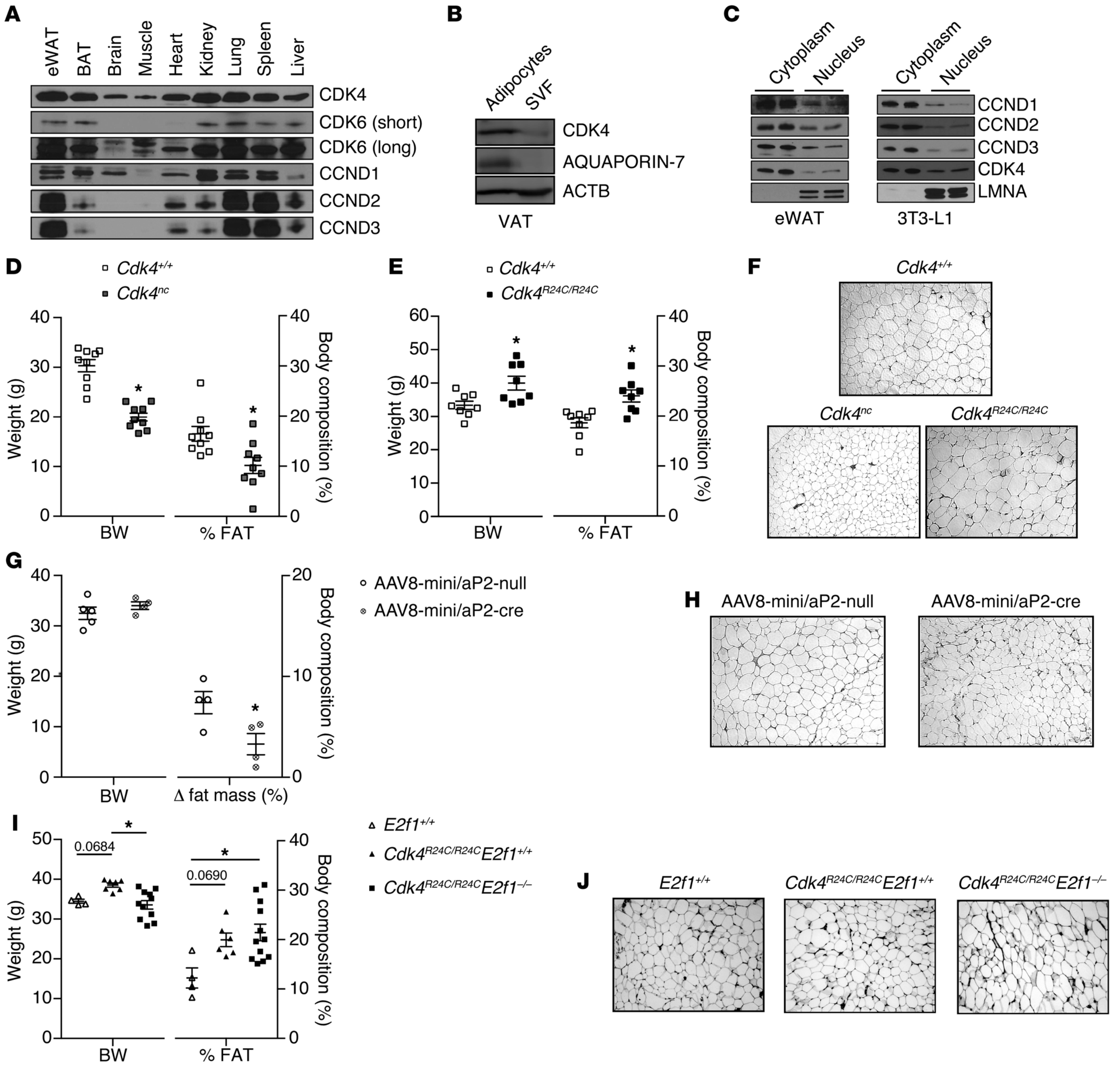

Figure 1. Positive correlation between CDK4 activity and WAT mass. (**A**) Expression levels of CCND1, CCND2, CCND3, CDK4, and CDK6 proteins in mouse eWAT, BAT, brain, muscle, heart, kidney, lung, spleen, and liver. Representative blot of several animals analyzed is shown. (**B**) CDK4 protein level in the SVF and mature adipocytes isolated from VAT. (**C**) Subcellular localization of CCND1, CCND2, CCND3, and CDK4 proteins in cytoplasm and nuclear fractions of eWAT and mature 3T3-L1 adipocytes. LMNA was used as a control for the nuclear fraction. (**B** and **C**) Representative blots out of 3 independent experiments are shown. (**D** and **E**) Body weight and percentage of fat mass of 20-week-old *Cdk4*$^{+/+}$ and *Cdk4*^{nc} mice (n = 9) (**D**) ~~and 30-week-old *Cdk4+/+* and *Cdk4R24C/R24C* mice (n = 8) (**E**) as obtained using EchoMRI technology. (**F**) H&E staining of eWAT sections from *Cdk4+/+*, *Cdk4nc*, and *Cdk4R24C/R24C* mice. (**G**) Body weight, Δ fat mass of 20-week-old *Cdk4flox/flox* mice infected with AAV8-mini/aP2-null (n = 5) or AAV8-mini/aP2-cre (n = 4) analyzed by EchoMRI technology (we show the difference between the percentage of fat before and the percentage of fat 3 weeks after infection). (**H**) H&E staining of eWAT sections from *Cdk4flox/flox* mice infected with AAV8-mini/aP2-null or AAV8-mini/aP2-cre. (**I**) Body weight and percentage of fat mass of 30-week-old *E2f1+/+* (n = 4), *Cdk4R24C/R24C E2f1+/+* (n = 6), and *Cdk4R24C/R24C E2f1−/−* mice (n = 12). (**J**) H&E staining of eWAT sections from~~ and 30-week-old *Cdk4*$^{+/+}$ and *Cdk4*$^{R24C/R24C}$ mice (n = 8) (**E**) as obtained using EchoMRI technology. (**F**) H&E staining of eWAT sections from *Cdk4*$^{+/+}$ (23 week old), *Cdk4*^{nc} (29 week old), and *Cdk4*$^{R24C/R24C}$ (54 week old) mice. (**G**) Body weight, Δ to fat mass of 14- to 16-week-old *Cdk4*$^{flox/flox}$ mice infected with AAV8-mini/aP2-null (n = 5) or AAV8-mini/aP2-cre (n = 4) analyzed by EchoMRI technology (we show the difference between the percentage of fat before and the percentage of fat 3 weeks after infection). (**H**) H&E staining of eWAT sections from 16-to 18-week-old *Cdk4*$^{flox/flox}$ mice infected with AAV8-mini/aP2-null or AAV8-mini/aP2-cre. (**I**) Body weight and percentage of fat mass of 30-week-old *E2f1*$^{+/+}$ (n = 4), *Cdk4*$^{R24C/R24C}$ *E2f1*$^{+/+}$ (n = 6), and *Cdk4*$^{R24C/R24C}$ *E2f1*$^{-/-}$ mice (n = 12). (**J**) H&E staining of eWAT sections from 33-week-old *E2f1*$^{+/+}$, *Cdk4*$^{R24C/R24C}$ *E2f1*$^{+/+}$, and *Cdk4*$^{R24C/R24C}$ *E2f1*$^{-/-}$ mice. Statistically significant differences were determined with unpaired 2-tailed Student's t tests (**D**, **E**, and **G**) or 1-way ANOVA followed by Tukey's multiple comparisons test (**I**). *$P < 0.05$. Original magnification, ×100.

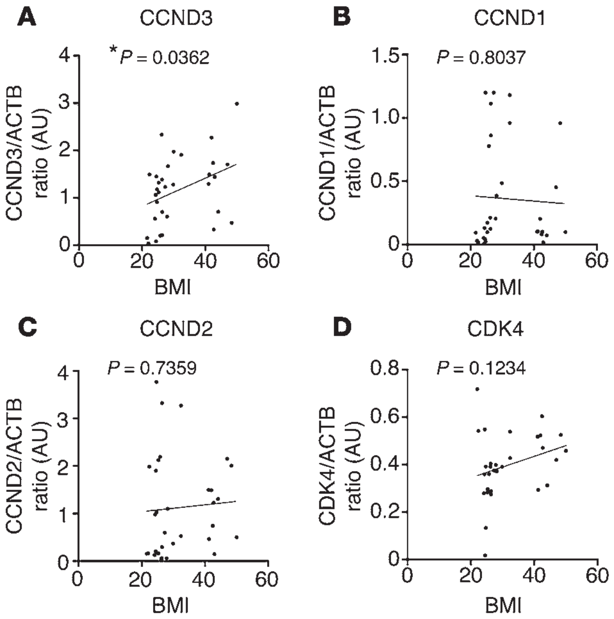

Figure 2. Positive correlation between CCND3 expression and human VAT mass. (**A**–**D**) Correlation between the CCND3/ACTB ratio (n = 32, Pearson's r = 0.3717, P < 0.05) (**A**), the CCND1/ACTB ratio (n = 32, Pearson's r = –0.04574, P < 0.05) (**B**), the CCND2/ACTB ratio (n = 32, Pearson's r = 0.06203, P < 0.05) (**C**), and the CDK4/ACTB ratio (n = 30, Pearson's r = 0.2875, P < 0.05) (**D**) and BMI in VAT of human subjects. Data are normalized to ACTB. *P < 0.05.

suggestion of a role of CDK4 in adipose tissue biology came from the finding that CDK4 and 2 D-type cyclins (CCND2 and CCND3) are highly expressed in epididymal WAT (eWAT) compared with the other tissues analyzed (Figure 1A). The high levels of expression of CCND3 in eWAT (Figure 1A and Supplemental Figure 1, A and B; supplemental material available online with this article; doi:10.1172/JCI81480DS1) are consistent with previous findings showing increased CCND3 expression during adipogenesis (15). Protein expression analysis in visceral adipose tissue (VAT) cellular fractions showed that CDK4 was better expressed in mature adipocytes compared with the stromal vascular fraction (SVF) (Figure 1B and Supplemental Figure 1C). Furthermore, CDK4 expression was also higher in differentiated 3T3-L1 adipocytes compared with nondifferentiated 3T3-L1 preadipocytes (Supplemental Figure 1C). Interestingly, the subcellular localization of CDK4 and CCND3 as well as of the other D-type cyclins revealed that these proteins are not only found in the nucleus; rather, they are mainly localized in the cytoplasm of adipocytes (Figure 1C and Supplemental Figure 1D), suggesting a role for CDK4 that is independent of the RB/E2F pathway in these cells. Moreover, since the duplication rate in mature adipocytes is low (16), these results suggested a novel cell-cycle independent role for CDK4. In order to analyze the participation of CDK4 in adipose tissue biology, we set to determine the phenotype of CDK4 mutant mice. The previously generated *Cdk4^neo/neo^* mice are diabetic and have impaired pancreatic β cell development and decreased insulin levels (11). Analysis of adipose tissue function in these mice would be confusing, since any observed effect could be secondary to insulin deficiency. We therefore used $Cdk4^{neo/neo}$ *Rip-Cre* ($Cdk4^{neo/neo;cre/cre}$; herein referred to as $Cdk4^{nc}$) mice that reexpress *Cdk4* in β cells and thus have normal insulin levels (13). We also used a mouse model of CDK4 hyperactivation, the R24C model. $Cdk4^{R24C/R24C}$ mice express a mutant CDK4 protein that is not sensitive to INK4a inhibitors (11) and is consequently more active. A first analysis showed that $Cdk4^{nc}$ mice had decreased body weight, whereas $Cdk4^{R24C/R24C}$ mice exhibited increased body weight compared with $Cdk4^{+/+}$ mice (Figure 1, D and E). Significant changes in WAT mass accounted for body weight variation. $Cdk4^{nc}$ and $Cdk4^{R24C/R24C}$ mice had decreased and increased WAT mass, respectively, as measured by EchoMRI (Figure 1, D and E, and Supplemental Figure 1, E and F). Changes in fat mass were consistent with variation in adipocyte size (Figure 1F and Supplemental Figure 1G). Overall, severe atrophy could be observed in fat pads from $Cdk4^{nc}$ mice, whereas $Cdk4^{R24C/R24C}$ mice developed adipose tissue hypertrophy (Supplemental Figure 1H).

To demonstrate that the effects of Cdk4 deletion in adipose tissue were cell autonomous, we used an approach involving systemic administration of adeno-associated viral vectors of serotype 8 (AAV8), which has been previously reported as leading to genetic engineering of white and brown adipocytes in adult mice and has very poor tropism for macrophages (17). We infected $Cdk4^{flox/flox}$ mice (Supplemental Figure 1I) with AAV8 vectors expressing the Cre recombinase under the control of the mini/aP2 adipose tissue–specific promoter (AAV8-mini/aP2-cre) or with the control vector (AAV8-mini/aP2-null). First of all, we determined the tissues that were infected by assessing the presence of viral genome (vg) using Cre PCR. The vg was only present in brown adipose tissue (BAT), eWAT, s.c. WAT, and liver, whereas we could not detect it in pancreas and muscle (Supplemental Figure 1J). Quantitative reverse-transcription–PCR (RT-qPCR) analysis showed a significant decrease of *Cdk4* mRNA in eWAT and s.c. WAT, whereas no changes were observed in liver and BAT (Supplemental Figure 1K). After 3 weeks, the systemic administration of AAV8-mini/aP2-cre triggered a decrease in fat mass gain; indeed, AAV8-mini/aP2-cre–infected mice gained significantly less fat mass (Figure 1G) and experienced a reduction in adipocyte size (Figure 1H). However, no differences were found in body weight and lean mass in $Cdk4^{flox/flox}$ mice infected with AAV8-mini/aP2-cre vector (Figure 1G and Supplemental Figure 1L). The use of this adipose tissue–specific *Cdk4* depletion model supports a cell-autonomous contribution for CDK4 in adipose tissue. Overall, these 3 models ($Cdk4^{nc}$, $Cdk4^{R24C/R24C}$, and $Cdk4^{flox/flox}$ mice infected with AAV8 mini/aP2-cre) clearly demonstrate a positive correlation between CDK4 activity and WAT mass/size.

E2F1, a known proproliferative downstream effector of CDK4, was previously shown to promote adipogenesis (16). Therefore, in order to determine whether adipocyte proliferation was not affected with the modulation of CDK4 activity, we generated $Cdk4^{R24C/R24C}$ $E2f1^{-/-}$ mice. No significant changes were observed in adiposity, adipocyte size, lean mass, or adipocyte proliferation as measured by Ki67 expression in $Cdk4^{R24C/R24C}$ $E2f1^{-/-}$ compared with $Cdk4^{R24C/R24C}$ $E2f1^{+/+}$ mice (Figure 1, I and J, and Supplemental Figure 1, M and N). These results demonstrate that when CDK4 is hyperactive, the deletion of E2f1 does not affect fat mass, mature adipocyte size, and proliferation. Because $Cdk4^{R24C/R24C}$ mice develop a wide spectrum of tumors (18, 19), we investigated

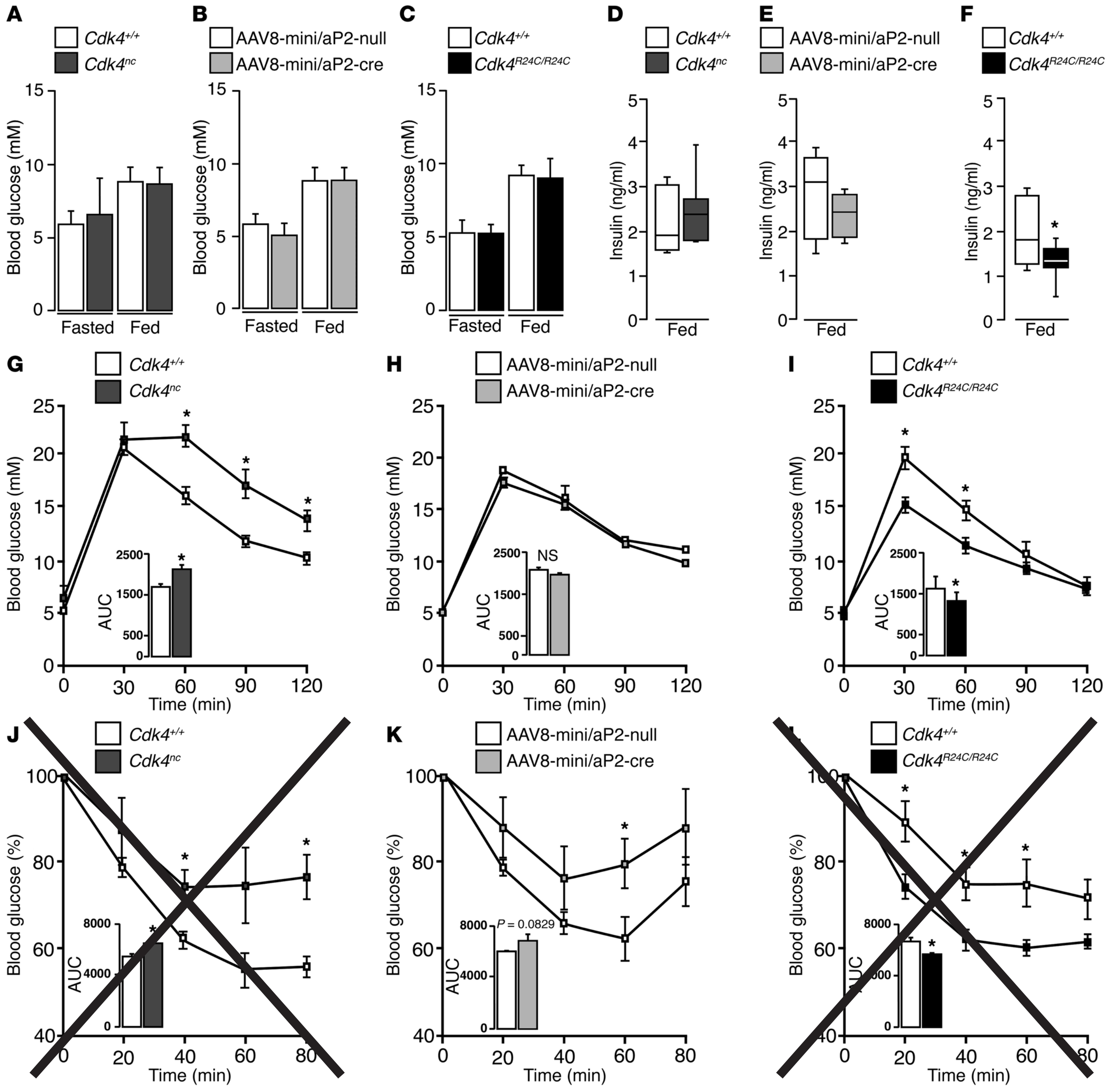

Figure 3. CDK4 promotes insulin sensitivity in vivo. ~~(**A**–**C**) Fasting glycemia in $Cdk4^{+/+}$ and $Cdk4^{nc}$ (n = 8) mice (**A**), $Cdk4^{flox/flox}$ infected with AAV8-mini/aP2-null or AAV8-mini/aP2-cre vectors (n = 5–4) (**B**), and $Cdk4^{+/+}$ and $Cdk4^{R24C/R24C}$ (n = 12) mice (**C**). (**D**–**F**) Fed serum insulin in $Cdk4^{+/+}$ and $Cdk4^{nc}$ (n = 7) (**D**), $Cdk4^{flox/flox}$ infected with AAV8-mini/aP2-null or AAV8-mini/aP2-cre vectors (n = 5–4) (**E**), and $Cdk4^{+/+}$ and $Cdk4^{R24C/R24C}$ mice (n = 8) (**F**). (**G**–**I**) GTT in $Cdk4^{+/+}$ and $Cdk4^{nc}$ (n = 7) (**G**), $Cdk4^{flox/flox}$ infected with AAV8-mini/aP2-null or AAV8-mini/aP2-cre (n = 5–4) (**H**), and $Cdk4^{+/+}$ and $Cdk4^{R24C/R24C}$ (n = 6) mice (**I**). (**J**–**L**) ITT in $Cdk4^{+/+}$ and $Cdk4^{nc}$ (n = 5) (**J**), $Cdk4^{flox/flox}$ infected with AAV8-mini/aP2-null or AAV8-mini/aP2-cre vectors (n = 5–4) (**K**), and $Cdk4^{+/+}$ and $Cdk4^{R24C/R24C}$ (n = 12) mice (**L**).~~ (**A**–**C**) Fasting glycemia of 17- to 21-week-old $Cdk4^{+/+}$ and $Cdk4^{nc}$ (n = 8) mice (**A**), 15- to 17-week-old $Cdk4^{flox/flox}$ mice infected with AAV8-mini/aP2-null (n = 5) or AAV8-mini/aP2-cre virus (n = 4) (**B**), and 33- to 36-week-old $Cdk4^{+/+}$ and $Cdk4^{R24C/R24C}$ mice (n = 12) (**C**). (**D**–**F**) Fed serum insulin of 21-week-old $Cdk4^{+/+}$ and $Cdk4^{nc}$ mice (n = 7) (**D**), 15- to 17-week-old $Cdk4^{flox/flox}$ mice infected with AAV8-mini/aP2-null (n = 5) or AAV8-mini/aP2-cre virus (n = 4) (**E**), and 29- to 33-week-old $Cdk4^{+/+}$ and $Cdk4^{R24C/R24C}$ mice (n = 8) (**F**). (**G**–**I**) GTT of 20- to 23-week-old $Cdk4^{+/+}$ and $Cdk4^{nc}$ mice (n = 7) (**G**), 15-to 17-week-old $Cdk4^{flox/flox}$ mice infected with AAV8-mini/aP2-null (n = 5) or AAV8-mini/aP2-cre (n = 4) (**H**), and 36-week-old $Cdk4^{+/+}$ and $Cdk4^{R24C/R24C}$ mice (n = 6) (**I**). (**J**–**L**) ITT of 24-week-old $Cdk4^{+/+}$ and $Cdk4^{nc}$ mice (n = 5) (**J**), 14- to 16-week-old $Cdk4^{flox/flox}$ mice infected with AAV8-mini/aP2-null (n = 5) or AAV8-mini/aP2-cre virus (n = 4) (**K**), and 30-week-old $Cdk4^{+/+}$ and $Cdk4^{R24C/R24C}$ mice (n = 5–11) (**L**). AUC for GTT and ITT was analyzed and is shown below the curves. Data were expressed as mean ± SEM. Statistically significant differences were determined with unpaired 2-tailed Student's t tests. $^*P < 0.05$.

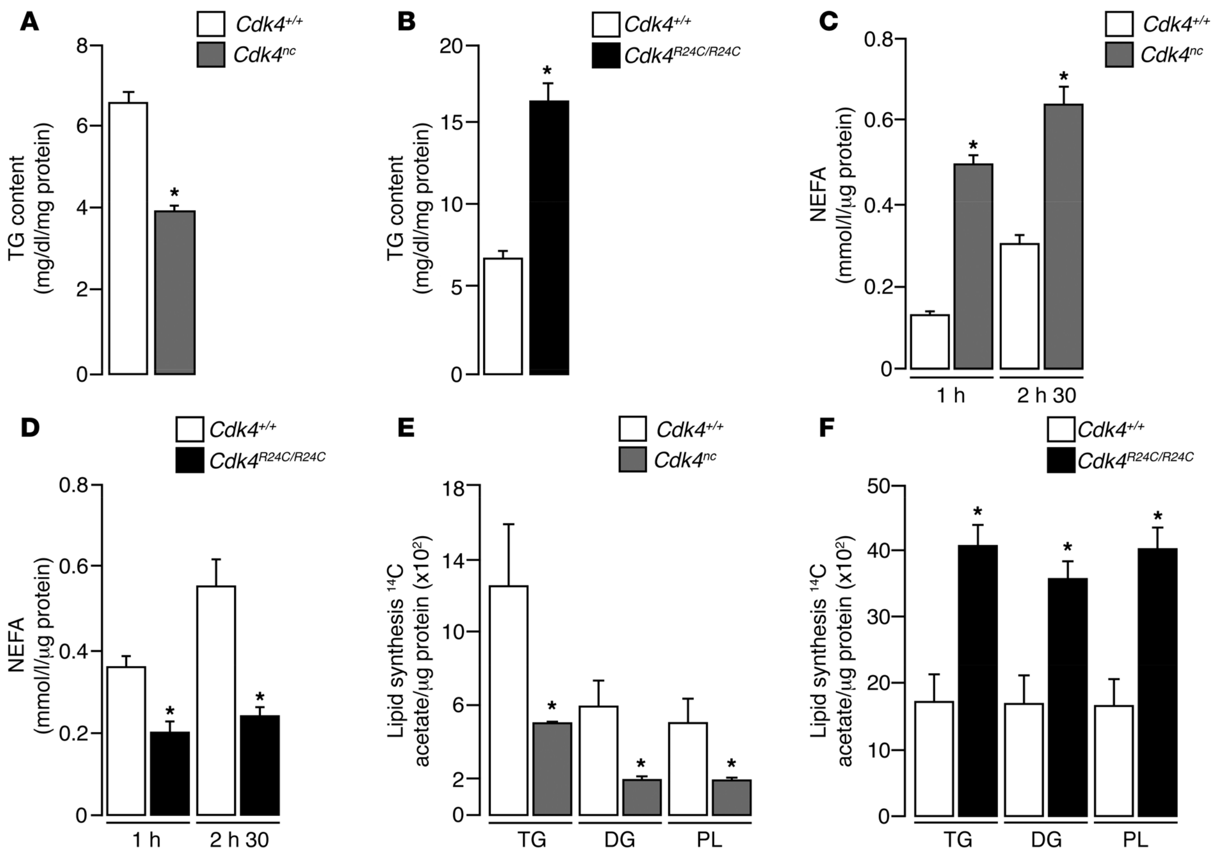

Figure 4. CDK4 represses lipolysis and is a positive modulator of lipogenesis. ~~(**A**) Quantification of TG content of eWAT from *Cdk4*$^{+/+}$ and *Cdk4*^{nc} mice (*n* = 3). (**B**) Quantification of TG content of eWAT of *Cdk4*$^{+/+}$ and *Cdk4*$^{R24C/R24C}$ mice (*n* = 3). (**C**) Rate of NEFA release in eWAT explants from fasting *Cdk4*$^{+/+}$ and *Cdk4*^{nc} mice (*n* = 3). (**D**) Rate of NEFA release in eWAT explants from fasting *Cdk4*$^{+/+}$ and *Cdk4*$^{R24C/R24C}$ mice (*n* = 6). (**E**) Ex vivo lipogenesis experiments in eWAT explants using labeled ^{14}C-acetate incorporation to detect TG, DG, and PL synthesis in *Cdk4*$^{+/+}$ and *Cdk4*^{nc} mice (*n* = 3). (**F**) Ex vivo lipogenesis experiments in eWAT explants from *Cdk4*$^{+/+}$ and *Cdk4*$^{R24C/R24C}$ mice. ^{14}C-acetate incorporation was used to detect TG, DG, and PL synthesis by TLC (*n* = 3).~~ (**A**) Quantification of triglyceride content of eWAT from 23-week-old *Cdk4*$^{+/+}$ and *Cdk4*^{nc} mice (*n* = 3). (**B**) Quantification of triglyceride content of eWAT from 27-week-old *Cdk4*$^{+/+}$ and *Cdk4*$^{R24C/R24C}$ mice (*n* = 3). (**C**) Rate of NEFA release in eWAT explants from 23-week-old fasting *Cdk4*$^{+/+}$ and *Cdk4*^{nc} mice (*n* = 3). (**D**) Rate of NEFA release in eWAT explants from 28-week-old fasting *Cdk4*$^{+/+}$ and *Cdk4*$^{R24C/R24C}$ mice (*n* = 6). (**E**) Ex vivo lipogenesis experiments in eWAT explants using labeled ^{14}C acetate incorporation to detect TG, DG, and PL synthesis of 22-week-old *Cdk4*$^{+/+}$ and *Cdk4*^{nc} mice (*n* = 3). (**F**) Ex vivo lipogenesis experiments in eWAT explants of 27-week-old *Cdk4*$^{+/+}$ and *Cdk4*$^{R24C/R24C}$ mice. ^{14}C-acetate incorporation was used to detect TG, DG, and PL synthesis by TLC (*n* = 3). Data are expressed as mean ± SEM. Statistically significant differences were determined with unpaired 2-tailed Student's *t* tests. $^{*}P < 0.05$.

to determine whether the WAT phenotype observed in these mice could be secondary to tumor development. We could not find any correlation between fat mass and tumor development. Indeed, all mice used in this study were tumor free (Supplemental Figure 1O). Moreover, tumor development was negatively correlated with fat mass in 60-week-old *Cdk4*$^{R24C/R24C}$ mice, proving that the increased WAT mass in these mice was not secondary to tumor formation (Supplemental Figure 1P).

CCND3 is positively correlated with WAT mass in human subjects. Our data suggesting the involvement of CCND3-CDK4 in adipose tissue was further supported by the positive correlation between CCND3 protein expression in visceral WAT samples from human subjects and their BMI (Figure 2A and Supplemental Figure 1, Q and R). No association was found for CCND1, CCND2, or CDK4 (Figure 2, B–D). These results confirmed a positive correlation between WAT mass and CCND3-CDK4 expression and activity and suggested that these proteins participate in WAT function.

~~*CDK4 promotes insulin sensitivity in vivo.*~~ *CDK4 regulates glucose homeostasis in vivo.* No differences in fasting and feeding glycemia were observed in *Cdk4*^{nc} or *Cdk4*$^{flox/flox}$ infected with AAV8-mini/aP2-cre vector and *Cdk4*$^{R24C/R24C}$ mice compared with their respective control mice (Figure 3, A–C). Insulin quantification in plasma showed, however, a significant decrease in *Cdk4*$^{R24C/R24C}$ mice, whereas no differences were observed in *Cdk4*^{nc} or *Cdk4*$^{flox/flox}$ mice infected with AAV8-mini/aP2-cre vector in fed conditions (Figure 3, D–F). Decreased insulin levels are indicative of either better insulin sensitivity or of a defect in insulin secretion by pancreatic β cells. ~~*Cdk4*^{nc} mice were glucose intolerant (Figure 3G) and cleared glucose at a slower rate than *Cdk4*$^{+/+}$ mice, a characteristic of insulin resistance (Figure 3J). We did not observe any significant differences in glucose tolerance tests (GTTs) in *Cdk4*$^{flox/flox}$ mice infected with AAV8-mini/aP2-cre vector; however, these mice had a trend toward insulin resistance compared with *Cdk4*$^{flox/flox}$ mice infected with AAV8-mini/aP2-null vector ($P = 0.0829$) (Figure 3, H and K). In contrast, *Cdk4*$^{R24C/R24C}$ mice were more glucose tolerant and insulin sensitive than *Cdk4*$^{+/+}$ mice (Figure 3, I and L). Together,~~

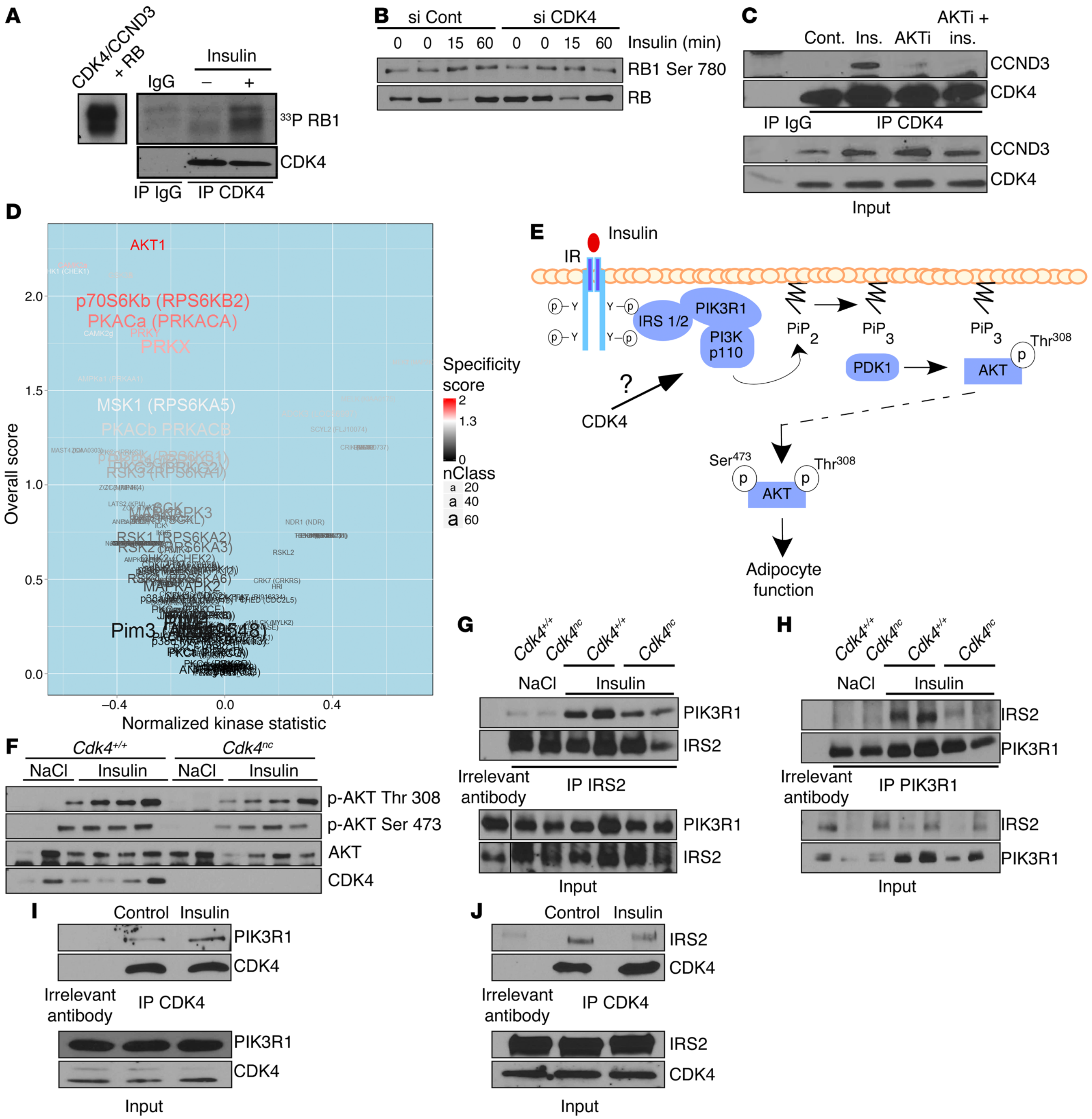

Figure 5. CDK4 is activated by insulin and translates insulin signaling in adipocytes. (**A**) CDK4 activity in vivo. SDS-PAGE autoradiography showing RB1 phosphorylation by CDK4 immunoprecipitated from 3T3-L1 mature adipocytes after insulin stimulation. The left panel shows RB1 phosphorylation by recombinant CDK4 used as a positive control. (**B**) Western blot analysis showing the inhibition of insulin-induced RB1 phosphorylation on Ser780 by CDK4 knockdown in mature 3T3-L1 adipocytes. (**C**) CCND3 and CDK4 association is increased upon insulin stimulation, but decreased upon cotreatment with insulin and AKT inhibitor in mature 3T3-L1 adipocytes. (**D**) ~~Volcano plot showing differences in putative kinase activities between control and *Cdk4*^{nc} mice injected (portal vein) with insulin for 3 minutes (*n* = 4).~~ Volcano plot showing differences in putative kinase activities between 22-week-old control and *Cdk4*^{nc} mice injected (portal vein) with insulin for 3 minutes (*n* = 4). Kinases with a positive kinase statistic show higher activity in *Cdk4*^{nc} samples compared with control samples, whereas kinases with negative kinase statistic show lower activity in *Cdk4*^{nc} samples compared with control samples. (**E**) Based on the upstream kinase activity (**D**) results and on the GeneGO analysis of the PamGene experiment, the putative role of CDK4 upstream of AKT in the insulin signaling pathway is represented. (**F**) ~~Immunoblot showing AKT phosphorylation on Thr308 and Ser473 in response to insulin injection (3 minutes) in fasted control and *Cdk4*^{nc} mice (*n* = 2 for NaCl and *n* = 4 for insulin treatment).~~ Immunoblot showing AKT phosphorylation on Thr308 and Ser473 in response to insulin injection (3 minutes) in 22-week-old fasted control and *Cdk4*^{nc} mice (*n* = 2 for NaCl and *n* = 4 for insulin treatment). (**G** and **H**) Coimmunoprecipitation experiments showing the interaction between endogenous IRS2 and PIK3R1. ~~IRS2 (**G**) and PIK3R1 (**H**) were immunoprecipitated and the presence of PIK3R1 (**G**) and IRS2 (**H**) was detected by Western blot analysis in *Cdk4*$^{+/+}$ and *Cdk4*^{nc} mice treated with insulin for 3 minutes (*n* = 2 for NaCl and *n* = 3 for insulin treatment).~~ IRS2 (**G**) and PI3Kp85A (**H**) were immunoprecipitated and the presence of PI3K p85A (**G**) and IRS2 (**H**) was detected

by Western blot analysis from 23-week-old *Cdk4*$^{+/+}$ and *Cdk4*^{nc} mice treated with insulin for 3 minutes. (n = 2 for NaCl and n = 3 for insulin treatment). (**I** and **J**) Coimmunoprecipitation experiments showing the interaction between endogenous PIK3R1 or IRS2 and CDK4. CDK4 was immunoprecipitated and the presence of PIK3R1 (n = 3) (**I**) and IRS2 (n = 1) (**J**) was detected by Western blot analysis in 3T3-L1 mature adipocytes treated with insulin. A representative Western blot is shown. Unless specified otherwise, all experiments are representative of 3 independent experiments.

~~these results show that CDK4 activity is positively correlated with insulin sensitivity.~~ *Cdk4*^{nc} mice were glucose intolerant (Figure 3G), with no significant differences in clearing glucose in response to insulin compared with *Cdk4*$^{+/+}$ mice (Figure 3J). We did not observe any significant difference in glucose tolerance test (GTT) in *Cdk4*$^{flox/flox}$ mice infected with AAV8-mini/aP2-cre virus; however, these mice had a trend toward insulin resistance compared with *Cdk4*$^{flox/flox}$ mice infected with AAV8-mini/aP2-null virus (P = 0.0829) (Figure 3, H and K). *Cdk4*$^{R24C/R24C}$ mice were more glucose tolerant (Figure 3I), with no differences in insulin sensitivity compared with *Cdk4*$^{+/+}$ mice (Figure 3L). These results suggest that glucose homeostasis in vivo is regulated by CDK4, at least in part by modulating adipose tissue function.

CDK4 represses lipolysis and stimulates lipogenesis. We previously showed the participation of CDK4 and CCND3 in adipogenesis. We proved that these proteins control the activity of the master regulator of adipocyte differentiation, PPARγ (14). In this study, we analyze the participation of CDK4 in the function of mature adipocytes. Lipids are mobilized from WAT through lipolysis, a breakdown of triglycerides (TG) into glycerol and free fatty acids (FFA) (20). As expected, changes in WAT mass were positively correlated with alterations in TG content in our mouse models: *Cdk4*^{nc} and *Cdk4*$^{R24C/R24C}$ mice had less and more TG in eWAT, respectively (Figure 4, A and B). Decreased TG content in *Cdk4*^{nc} eWAT was likely the result of an imbalanced lipogenesis/lipolysis ratio. Indeed, treatment of fully differentiated 3T3-L1 adipocytes with a chemical CDK4 inhibitor (PD0332991) (21) resulted in the delipidation of these adipocytes and in a 40% decrease in their TG content (Supplemental Figure 2, A and B). Lipolysis experiments in eWAT explants from mice revealed a 4-fold increase of nonesterified fatty acid (NEFA) release in *Cdk4*^{nc} eWAT compared with *Cdk4*$^{+/+}$ eWAT (Figure 4C). Similarly, glycerol release was also increased in these mice (Supplemental Figure 2C). Conversely, *Cdk4*$^{R24C/R24C}$ eWAT showed significantly decreased NEFA (Figure 4D) and glycerol release (Supplemental Figure 2D), which suggested impaired lipolysis. Interestingly, eWAT explants from *Cdk4*^{nc} mice, in addition to increased lipolysis, had reduced lipogenesis, as measured by acetate incorporation into the distinct TG, diacyclycerols (DG), and phospholipid (PL) lipid fractions (Figure 4E). On the other hand, eWAT explants from mice expressing the hyperactive *Cdk4*^{R24C} allele showed increased lipogenesis (Figure 4F). However, we could not detect any differences in liver TG content in *Cdk4*$^{+/+}$, *Cdk4*^{nc}, and *Cdk4*$^{R24C/R24C}$ mice (Supplemental Figure 2, E and F). This validated a positive correlation between CDK4 activity and lipogenesis and a negative correlation between CDK4 activity and lipolysis. Strikingly, these are exactly the effects of insulin in adipocytes, suggesting that CDK4 could mediate the effects of insulin in adipose tissue.

CDK4 is activated by insulin and mediates insulin signaling in adipocytes. We wanted next to uncover the mechanisms of CDK4 activation following insulin stimulation in adipocytes. CDK4 activity was stimulated by insulin in differentiated 3T3-L1 cells, as suggested by increased phosphorylation of PIK3R1 by immunoprecipitated CDK4 (Figure 5A). Similar results were observed when eWAT from insulin-treated mice was used (Supplemental Figure 3A). Interestingly, CDK4 knockdown (Figure 5B) or treatment of 3T3-L1 adipocytes with PD0332991 (Supplemental Figure 3B) abrogated the effects of insulin on PIK3R1 phosphorylation. Furthermore, association of CDK4 with its regulatory subunit CCND3 was dependent on the insulin signaling pathway, since AKT inhibition abolished this association (Figure 5C).

Next, we addressed how CDK4 could participate in the insulin-signaling pathway in adipocytes. Since the insulin signaling cascade is dependent on the rapid activation of a series of tyrosine and serine/threonine protein kinases (STKs), we used a new technology developed by PamGene to determine differential global kinase activity in *Cdk4*^{nc} and control mice in response to insulin. We used arrays that consisted of 140 immobilized serine/threonine-containing peptides that are targets of most known kinases (STK PamChips) (22). These chips were incubated with lysates prepared from eWAT from *Cdk4*^{nc} or control mice injected with insulin. The same experiment was performed using lysates from cells treated with PD0332991 and stimulated with insulin. Peptides whose phosphorylation varied significantly between the control and *Cdk4*^{nc}-treated mice or between the control and PD0332991-treated samples (Supplemental Figure 3, C and D) were indicative of differential specific kinase activities. Putative upstream kinase analysis underscored significant differences in the AKT pathway (Figure 5D and Supplemental Figure 3, E and F). This suggested that CDK4 activity played a role upstream of AKT, as indicated in Figure 5E. Western blot analyses further proved that AKT activity, as measured by phosphorylation in Ser473 and Thr308, was decreased in *Cdk4*^{nc} mice in response to insulin (Figure 5F). Similarly, chemical inhibition of CDK4 also attenuated AKT signaling in 3T3-L1 adipocytes (Supplemental Figure 3G).

Table 1. CDK consensus sites in mouse IRS1, IRS2, PDKP1, and PIK3R1

Protein	Site position	Sequence	CDK4 Interaction
PIK3R1	Thr86	TPKP	+
PDPK1	Thr357	TPPP	–
	Thr521	TPNP	
IRS1	Ser1209	SPRR	+
IRS2	Ser388	SPGP	+
	Thr576	TPAR	
	Ser980	SPKP	
	Ser1004	SPYP	
	Ser1226	SPMR	

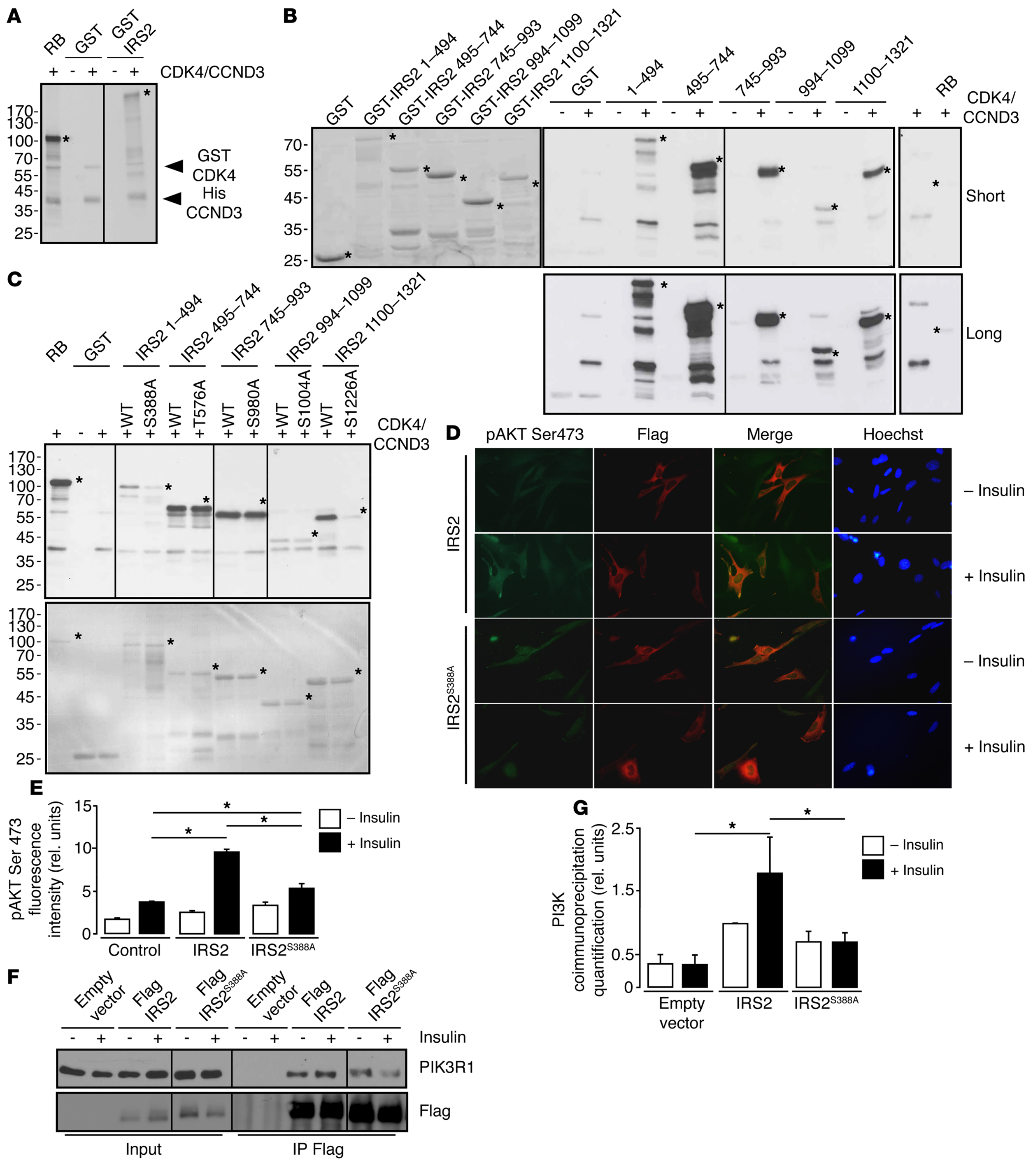

Figure 6. CDK4 phosphorylates IRS2. (**A**) CCND3-CDK4 complex directly phosphorylates full-length GST-IRS2 in vitro (n = 3). (**B**) In vitro phosphorylation of GST-IRS2 fragments (1–494aa, 495–744aa, 745–993aa, 994–1099aa, 1100–1321aa) by CCND3/CDK4. Left panel, SDS-PAGE stained with Coomassie blue for the loading control. Middle panels, autoradiography of the SDS-PAGE gels containing the different GST-IRS2 fragments, incubated with CCND3/CDK4. Right panel, RB1 recombinant protein was used as a positive control (n = 3). (**C**) Defective IRS2^{S388A} and IRS2^{S1226A} phosphorylation by CCND3-CDK4. Upper panel, autoradiography; lower panel, SDS-PAGE gel stained with Coomassie blue for the loading control. (n = 2). (**D**) Decrease in pAKT Ser473 phosphorylation in Flag-IRS2^{S388A} electroporated *Irs2*$^{-/-}$ cells upon insulin stimulation, compared with the WT Flag-IRS2–transfected cells (n = 3). Original magnification, ×400. (**E**) Quantification of pAKT Ser473 fluorescence intensity for untransfected, Flag-IRS2–transfected, and Flag-IRS IRS2^{S388A} electroporated *Irs2*$^{-/-}$ preadipocytes was performed with ImageJ software (http://imagej.nih.gov/ij/). At least 100 cells were quantified per condition. (**F**) Representative Western blot analysis showing impaired interaction between PIK3R1 and Flag-IRS2^{S388A} mutant after insulin stimulation compared with cells transfected with Flag-IRS2 in 293T cells. (**G**) Quantification of the blot shown in **F**. A representative Western blot is shown. Data are expressed as mean ± SEM. Statistically significant differences were determined with 2-way ANOVA followed by Tukey's multiple comparisons test (**E**–**G**). *P < 0.05.

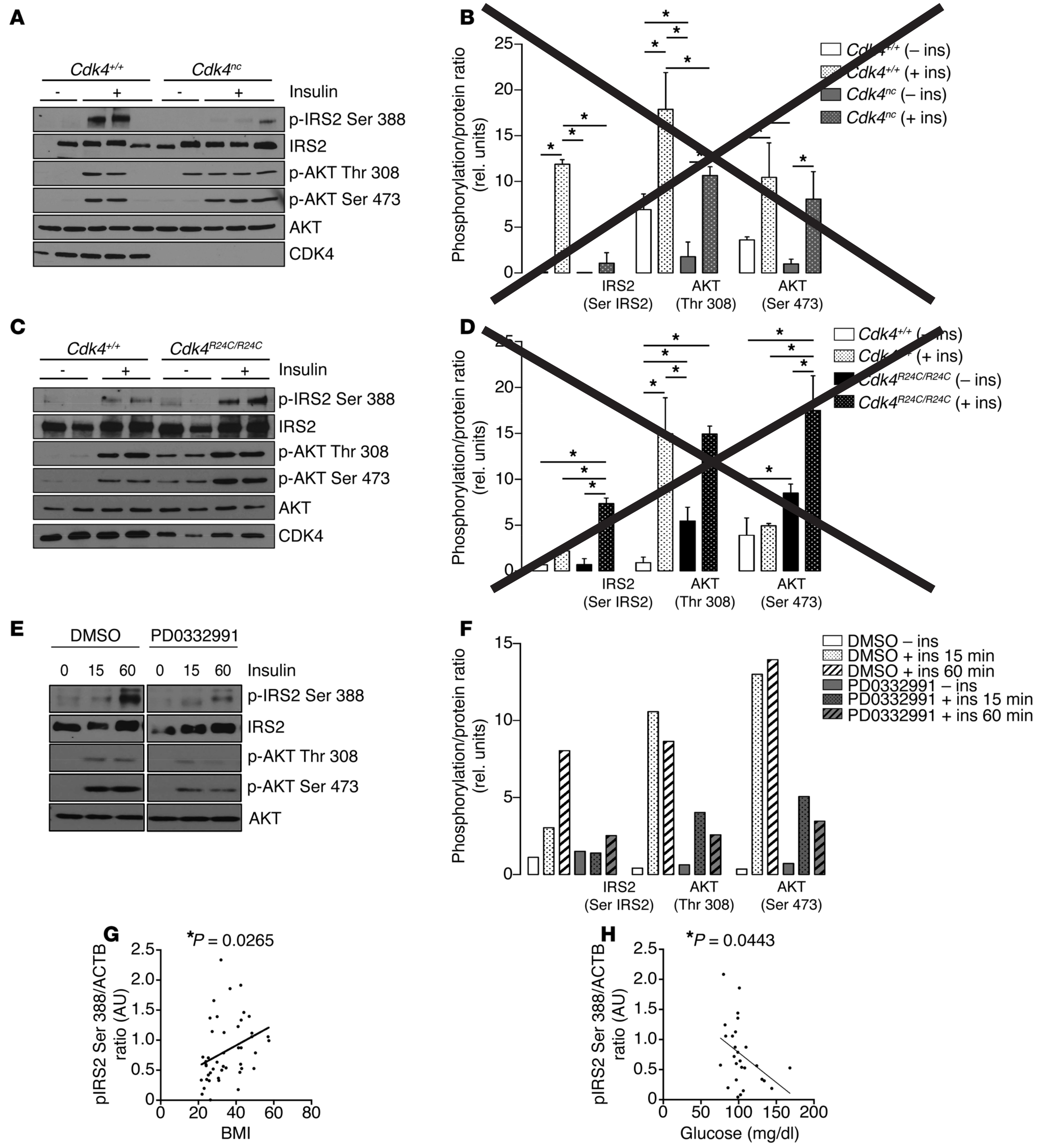

Figure 7. CDK4 phosphorylates in vivo the IRS2 protein at the Ser388. ~~(**A–D**) Immunoblot analysis of IRS2 phosphorylation on Ser388 and AKT phosphorylation on Thr308 and Ser473 in control and *Cdk4*^{nc} (*n* = 2 starved/5 insulin for *Cdk4*$^{+/+}$ and *n* = 2 starved/5 insulin for *Cdk4*^{nc}) mice (**A**). (**B**) Quantification of the blot shown in **A** using ImageJ software. *Cdk4*$^{R24C/R24C}$ (*n* = 2 starved/3 insulin for both *Cdk4*$^{+/+}$ and *Cdk*$^{R24C/R24C}$).~~ (**A–D**) Immunoblot analysis of IRS2 phosphorylation on Ser388 and AKT phosphorylation on Thr308 and Ser473 of 27-week-old control and *Cdk4*^{nc} mice (*n* = 2 starved/3 insulin for *Cdk4*$^{+/+}$ and *n* = 2 starved/3 insulin for *Cdk4*^{nc}) (**A**). (**B**) Quantification of the blot showed in **A** using ImageJ software. Please note that the mouse in lane 5 was not considered for analysis, since this control mouse was not responsive to insulin. *Cdk*$^{R24C/R24C}$ (*n* = 2 starved/3 insulin for both *Cdk4*$^{+/+}$ and *Cdk*$^{R24C/R24C}$) (40 weeks old). (**C**) Mice were treated for 50 minutes with insulin. (**D**) Quantification of the blot shown in **C** using ImageJ software. (**E**) Immunoblot analysis of IRS2 phosphorylation on Ser388 and AKT phosphorylation on Thr308 and Ser473 in 3T3-L1 mature adipocytes during a time course insulin stimulation with or without PD0332991 (*n* = 1). (**F**) Quantification of the blot shown in **E** using ImageJ software. (**G**) Correlation between the pIRS2 Ser388/ACTB ratio in VAT and the BMI of the subjects (*n* = 45, Pearson's *r* = 0.3307, $P < 0.05$). (**H**) Correlation between the pIRS2 Ser388/ACTB ratio in VAT and the glycemia of the subjects (*n* = 27, Pearson's *r* = –0.3900, $P < 0.05$). Data are expressed as mean ± SEM. Statistically significant differences were determined with 2-way ANOVA followed by Tukey's multiple comparisons test (**B–D**). *$P < 0.05$.

This further supports the hypothesis that CDK4 regulates insulin signaling upstream of AKT. Upon insulin stimulation, the intrinsic tyrosine kinase domain of the insulin receptor leads to receptor autophosphorylation at tyrosine residues. The subsequent recruitment and phosphorylation of insulin receptor substrate 1 (IRS1) and IRS2 are the pivotal events that, in turn, activate the downstream PI3K-PDK1-AKT axis to regulate lipogenesis and lipolysis in adipocytes (23). We therefore investigated whether CDK4 regulated the insulin-signaling pathway by facilitating the recruitment of IRS into PIK3R1, the PI3K subunit p85a. We found that *Cdk4* deletion greatly impaired the ability of IRS2 to bind with PIK3R1 in response to insulin stimulation (Figure 5, G and H). Furthermore, CDK4 was recruited to PIK3R1 (Figure 5I) and IRS2 (Figure 5J) complexes in adipocytes. However, we only detected an increase of interaction in response to insulin between PIK3R1 and CDK4.

IRS2 is a substrate of CDK4. Interestingly, in silico analysis highlighted the presence of CDK4 consensus phosphorylation sites in the p85A subunit of PI3K (PIK3R1), phosphoinositide-dependent kinase 1 (PDK1), IRS1, and IRS2 (Table 1). However, in vitro kinase assays showed no phosphorylation of PIK3R1, or PDPK1 by CDK4 (data not shown). In vitro kinase assays showed, however, that CDK4 could phosphorylate recombinant glutathione S-transferase–purified (GST-purified) IRS2 protein (Figure 6A). IRS2 contains 5 CDK4 consensus sites distributed along the protein (Ser388, Thr576, Ser980, Ser1004, and Ser1226) (Table 1 and Supplemental Figure 4A). Site-directed mutagenesis (serine to alanine) and protein truncation approaches helped us to map the CDK4 phosphorylation sites of IRS2 at Ser388 and Ser1226 (Figure 6, B and C). Interestingly, these 2 potential phosphorylation sites are highly conserved through evolution (Supplemental Figure 4B).

We next evaluated the functional relevance of $IRS2^{S388A}$ and $IRS2^{S1226A}$ mutants that cannot be phosphorylated by CDK4. Rescue of IRS2 activity in $Irs2^{-/-}$ preadipocytes with ectopic expression of WT IRS2 resulted in the restoration of insulin signaling as assessed by immunofluorescence staining of AKT phosphorylation (Figure 6, D and E). In contrast, $IRS2^{S388A}$ mutants, which cannot be phosphorylated by CDK4, could not restore insulin signaling in these cells (Figure 6, D and E). No significant phenotype was observed for $IRS2^{S1226A}$ mutants (data not shown). Moreover, $IRS2^{S388A}$ mutants were not recruited to PIK3R1 protein complexes upon insulin stimulation when ectopically expressed in 293T cells (Figure 6, F and G). This demonstrated that the phosphorylation of IRS2 on Ser388 by CDK4 is essential for its activity.

CDK4 regulates insulin signaling in vivo via $IRS2^{Ser388}$ phosphorylation. To determine the potential roles of CDK4 on IRS2 phosphorylation, we generated a phosphospecific antibody to Ser388 of IRS2 that we validated by in vitro CDK4 kinase assay (Supplemental Figure 5A). IRS2 Ser388 was highly phosphorylated in the adipose tissue of $Cdk4^{+/+}$ mice after 50 minutes of insulin stimulation (Figure 7, A and B). ~~This phosphorylation was almost abrogated in the adipose tissue of insulin-treated $Cdk4^{nc}$ mice. Moreover, decreased IRS2 Ser388 phosphorylation resulted in impaired insulin signaling pathways, as demonstrated by reduced AKT phosphorylation (Figure 7, A and B). In sharp contrast, CDK4 hyperactivity, as observed in $Cdk4^{R24C/R24C}$ mice, resulted in a robust increase in IRS2 Ser388 phosphorylation (Figure 7, C and D). Consequently, AKT phosphorylation was also increased (Figure 7, C and D).~~ This phosphorylation was almost not detectable in the adipose tissue of insulin-treated $Cdk4^{nc}$ mice. Moreover, decreased IRS2 Ser 388 phosphorylation showed a tendency to impair the insulin-signaling pathway, as demonstrated by reduced AKT phosphorylation (Figure 7, A and B). In sharp contrast, CDK4 hyperactivity, as observed in $Cdk4^{R24C/R24C}$ mice, resulted in an increase in IRS2 Ser 388 phosphorylation (Figure 7, C and D). In addition, AKT phosphorylation was also augmented (Figure 7, C and D). Chemical inhibition of CDK4 also resulted in the abrogation of both IRS2 Ser388 and AKT phosphorylations (Figure 7, E and F). ~~From these results, we can conclude that this newly identified site in IRS2, which is phosphorylated by CDK4, maintains the activation of the insulin signaling pathway.~~ From these results, we can hypothesize that this newly identified site in IRS2, which is phosphorylated by CDK4, maintains the insulin signaling pathway that is activated. To further investigate the status of IRS2 Ser388 in type 2 diabetic mouse models, such as *db/db* mice, that are known to be hyperinsulinemic, we analyzed this phosphorylation in the basal state and upon insulin stimulation in adipose tissue. The *db/db* mice have a tendency toward an increased IRS2 Ser388 phosphorylation under basal conditions, compared with *db/+* mice ($P = 0.0511$) (Supplemental Figure 5B). However, insulin-resistant *db/db* mice did not show increased IRS2 Ser388 phosphorylation upon insulin stimulation (Supplemental Figure 5B). Most important was the finding that IRS2 Ser388 phosphorylation in human visceral WAT samples was positively correlated with the BMI of the subjects (Figure 7G and Supplemental Figure 5, C and D). Interestingly, we found a negative correlation between IRS2 Ser388 phosphorylation and fasting glucose in human subjects. This further advocates for a role of CDK4 in both adipose tissue biology and glucose homeostasis (Figure 7H and Supplemental Figure 5E).

Discussion

We showed throughout this study that the cell-cycle regulatory kinase CDK4 is a key regulator of adipocyte function. The participation of this kinase in the control of proliferation through the control of the activity of E2F transcription factors has been extensively studied (24). The results of our study provide 3 lines of evidence that CDK4 acts independently of E2F to regulate adipocyte function. First, we discovered a role of the cell-cycle kinase CDK4 in the control of the insulin-signaling pathway. CDK4, through phosphorylation of IRS2, maintains insulin action in adipocytes. This is consistent with the phenotypes of genetic CDK4 mouse models. Indeed, adipose tissue from $Cdk4^{nc}$ mice has decreased lipogenesis as well as increased lipolysis. In contrast, mice that express hyperactive $CDK4^{R24C}$ exhibit decreased lipolysis and increased lipogenesis in WAT. These findings place CDK4 at the initiation of the insulin-triggered adipocyte-signaling pathway. In line with a role of circulating lipids in controlling glucose homeostasis, $Cdk4^{nc}$ mice showed decreased glucose tolerance whereas $CDK4^{R24C}$ mice showed increased glucose tolerance. Importantly, no significant differences in insulin sensitivity were observed in the $Cdk4^{nc}$ and $CDK4^{R24C}$ mice, pointing to a potential role of CDK4 in other metabolic organs that could also be affected in our mouse models.

~~We~~ In addition, we show in this study a function of CDK4,

that of a mediator of insulin signaling. Indeed, we show that the effects of CDK4 in adipocytes are independent of E2F activity and, therefore, most likely independent of the control of the cell cycle. E2F1 is the most studied member of the E2F family. E2F1 has numerous metabolic functions, such as the participation in adipose tissue metabolism through the transcriptional regulation of the master adipogenic factor PPARγ during early stages of adipogenesis (16). Here, we demonstrate, by generation of *Cdk4*$^{R24C/R24}$ *E2f1*$^{+/+}$ and *Cdk4*$^{R24C/R24C}$ *E2f1*$^{-/-}$ mice, that CDK4 has E2F1-independent functions in mature adipose tissue. Indeed, the genetic deletion of E2F1 in the R24C background does not affect adiposity or adipocyte proliferation (Figure 1, I and J, and Supplemental Figure 1N).

Based on our results, we propose that CDK4 is integrated in the insulin-signaling pathway as follows. In response to insulin, the canonical cascade of events is elicited. This includes the sequential activation of IR, IRS1-2, PI3K, PDPK1, and AKT. AKT then activates CDK4 (because inhibition of AKT blocks CDK4 activation), which then phosphorylates IRS2, creating a positive feedback loop. The activation of CDK4 by AKT is likely an indirect event. Several studies previously reported that AKT phosphorylates and inhibits p21 and p27, which are both CDK4 inhibitors (25, 26).

Interestingly, IRS proteins are also involved in the activation of several growth factor receptor pathways other than the insulin receptor pathway, such as the IGF 1 receptor (IGF1R) pathway (27). The prooncogenic activities of IGF1R (26) are mediated by its downstream effectors, IRS1 and IRS2. IRS proteins transduce mitogenic, antiapoptotic, and antidifferentiation signals to the cell, mainly through the PI3K–AKT module (28). Although antioncogenic synergistic effects have been observed using either CDK4 and IGF1R inhibitors or CDK4 and PI3K inhibitors, no crosstalk between both pathways has been described (29–32). The CDK4/CDK6 inhibitor (PD0332991, palbociclib) has been approved for the treatment of breast cancer (33). Two other CDK4/CDK6 inhibitors, LY-2835219 (also known as abemaciclib) and LEE011 (also known as ribociclib), are also currently in advanced stages of clinical trials (34). Interestingly, it has previously been reported that a major enzyme of de novo lipogenesis, the fatty acid synthase (FASN), is increased in numerous cancers, including breast cancer (35). The activity of FASN is known to be stimulated by insulin through the PI3K/AKT pathway, and here, we demonstrate that CDK4 is a key effector of insulin, thus promoting de novo lipid synthesis. Based on our findings, we can speculate that CDK4, through phosphorylation and regulation of IRS activity, could simultaneously sustain de novo lipid synthesis and the oncogenic activity of the aforementioned pathways in transformed cells.

The second major finding of our study is the discovery of a residue in IRS2 that is phosphorylated by CDK4 in response to insulin. A large number of publications previously focused on the effects of IRS1 and IRS2 phosphorylation on the insulin-signaling pathway. Both positive and negative phosphorylation sites finely regulate IRS1 and IRS2 activity and are a paradigm of the flexibility of insulin and IGF signaling (36). The final serine/threonine phosphorylation state of IRS proteins is a consequence of the combined action of several kinases that are activated by different pathways in a spaciotemporal manner. Multiple site phosphorylation of these proteins by distinct kinases, such as JNK (37), GSK3 (38), ERK1, or mTOR (39), provides a large number of combinations of phosphorylating events that generate a very complex network (40). We show that CDK4 phosphorylates IRS2 at the new Ser388 site. ~~Moreover, we show that this phosphorylation renders IRS2 more active.~~ Moreover, our observations suggest that this phosphorylation renders IRS2 more active. Our results add more complexity to the understanding of the physiology of the phosphorylation of the IRS proteins. Phosphorylation by CDK4 may also have an impact on the phosphorylation of IRS2 by other kinases; however, the relative contribution of CDK4 to the final activation or inhibition of IRS compared with other kinases and the identification of which serine/threonine residues are the most critical in regulating IRS function in response to insulin remain to be elucidated. Similarly, we still do not understand why CDK4 activates IRS2 whereas AKT inhibits it through a negative feedback loop. The same stimulus, insulin, triggers concomitantly inhibitory and stimulatory phosphorylations in IRS. The fine regulation of these positive versus negative phosphorylation events requires further investigation. Interestingly, we were able to observe the stimulation of IRS2 Ser388 phosphorylation with insulin not only in adipocytes, but also in other cell types, such as C2C12 myotubes and primary hepatocytes. IRS2 Ser388 phosphorylation could also be detected in Min6 cells, but was not so insulin responsive. As shown in Supplemental Figure 6, A–C, upon insulin stimulation, IRS2 Ser388 phosphorylation was stronger after 1 hour of treatment, which is in agreement with our findings in mature adipocytes. These results open interesting perspectives into the contribution of CDK4 to IRS2 Ser388 phosphorylation in other insulin-sensitive tissues; CDK4 could, for instance, participate in the control of de novo lipid synthesis in liver upon insulin stimulation.

RNAi-mediated depletion of CDK6, the CDK4 ortholog, suggests that this kinase is also able to phosphorylate IRS2 at Ser388 in mature adipocytes upon insulin stimulation, but to a lesser extent (Supplemental Figure 6D). The effects of CDK6 on IRS2 in other tissues remain to be studied.

Defects in insulin action and insulin secretion are both features of type 2 diabetes. In line with previous publications reporting that CDK4 regulates β cell growth (11) and insulin secretion in β cells (41), it would be interesting to explore the relative contribution of this IRS2 Ser388 phosphorylation in β cell function. The involvement of IRS2 in the pathogenesis of type 2 diabetes is highlighted by the phenotype of *Irs2*$^{-/-}$ mice. Indeed, these animals develop type 2 diabetes with impaired peripheral insulin signaling and pancreatic β cell function without compensation by IRS1 (42).

Third, the importance of our findings goes beyond the control of adipocyte biology in normal physiology. Here, we also report a significant ($P = 0.0362$) correlation between CCND3 expression and BMI in human subjects. Our model is further supported by the observed increase in the phosphorylation of the Ser388 of IRS2 in human obese subjects (BMI > 27). Insulin resistance is a major feature in various metabolic disorders, such as obesity and type 2 diabetes. We show here an inverse correlation of IRS2 Ser388 levels in VAT with the blood glucose levels from the subjects (Figure 7H). This strongly supports the notion that this phosphorylation participates in glucose homeostasis in humans.

In conclusion, our results demonstrate that CDK4 is a regulator of adipocyte insulin signaling. By combining experimental data from cellular and mouse models and data obtained using human

samples, our study provides insights into the complex pathogenesis of obesity and insulin resistance.

Methods

Materials. All experiments with the CDK4 inhibitor (PD0332991, Azasynth Co.) were done using 1 μM of PD0332991 in mature 3T3-L1 adipocytes. All chemicals, unless stated otherwise, were purchased from Sigma-Aldrich. Actrapid human recombinant insulin was purchased from Novo Nordisk Pharma SA. AKT inhibitor (catalog 124017) was purchased from Calbiochem and used at 10 μM for 30 minutes. ^{14}C-acetate, and γ-^{33}P-ATP were purchased from PerkinElmer.

Animals. The generation of *Cdk4*^{nc} and *Cdk4*$^{R24C/R24C}$ mice has been previously described (11, 13). The 8- to 12-week-old male db/+ and db/db as well as C57BL/6J (B6) mice were obtained from Janvier. *E2f1*$^{+/+}$ and *E2f1*$^{-/-}$ mice (B6;129S4-E2f1tm1 Meg/J) were purchased from The Jackson Laboratory. *Cdk4*$^{flox/flox}$ mice were generated for this study in collaboration with Cyagen Biosciences. The targeting vector included a Neo resistance cassette flanked by FRT sites as well as CRE-dependent lox P sites in introns 1 and 8 (Supplemental Figure 1I).

C57BL/6 embryonic stem cells were used for gene targeting, and the positive cells were bred into albino B6 female mice. This strategy allowed us to have a pure B6 background. *Cdk4*$^{flox/+}$ mice were then crossed with mice expressing Flp recombinase (B6.Cg-Tg[Pgk1-FLPo]10Sykr/J) in order to remove the Neo resistance cassette. With one subsequent cross with B6 animals, the Flp transgene was removed and the obtained *Cdk4*$^{flox/+}$ mice were then intercrossed in order to generate the *Cdk4*$^{flox/flox}$ mice used in this study.

Animals were maintained in a temperature-controlled animal facility with a 12-hour light/12-hour dark cycle and had access to food and water according to the Swiss Animal Protection Ordinance (OPAn). Only male animals were used in this study. For the GTT, mice were starved for 16 hours and then injected i.p. with glucose (2 g/kg). Tail vein blood glucose was measured at the indicated times. For the insulin tolerance test (ITT), 6-hour–fasted mice were injected i.p. with 0.75 U/kg insulin and tail vein blood glucose was then measured at the indicated times. For the in vivo insulin-stimulation assay, mice were fasted overnight and injected in the portal vein or i.p. with 0.75 U/kg insulin or an equal volume of saline. After 3 or 50 minutes, the mice were sacrificed via cervical dislocation. For insulin level measurements, tail vein blood was collected under fed conditions 2 hours after the beginning of the 12-hour dark cycle.

Plasmid constructs and mutagenesis. pDONR-IRS2 was subcloned from pBS mouse IRS-2 (Addgene plasmid catalog 11372) (43) and generated using the pDONR221 vector of Gateway Cloning Technology (Invitrogen). Flag-IRS2 and GST-IRS2 were obtained using the pDEST pCMV14-3XFlag and pGEX-2T vectors of Gateway Cloning Technology starting from the above-described pDONR-IRS2 constructs. A similar strategy was used to obtain the truncated versions of GST-IRS2. The Flag-PIK3R1 and Flag-PDPK1 plasmids were obtained from the Montpellier Genomic Collection (MGC). pDONR-hRB 379–928aa was subcloned from pCMV human RB1 and generated using the pDONR221 vector of Gateway Cloning Technology. pGEX-2T hRB 379–928aa was obtained using the pDEST pGEX-2T from Gateway Cloning Technology. The different serine-to-alanine mutants of GST-IRS2 were generated using a QuikChange Site-Directed Mutagenesis Kit (Stratagene).

Cell culture. 3T3-L1 and 293T were obtained from ATCC. *Irs2*$^{-/-}$ cells were cultured in DMEM with 10% FBS (PAA Laboratories) in 5% CO_2 in an incubator set at 37°C. Two days after reaching confluence, 3T3-L1 cells were differentiated with DMEM, 10% FBS, 0.5 mM 3-isobutyl-1methylxanthine (IBMX), 1.7 μM insulin, 1 μM dexamethasone, and 1 μM rosiglitazone for 2 days. From day 3 onward, the cells were incubated with DMEM, 10% FBS, and 10 μg/ml insulin, and the medium was changed every 2 days until day 8 of differentiation. 3T3-L1 mature adipocytes were maintained in medium containing FBS only. For insulin (100 nM) or isoproterenol (100 nM) treatments, fully differentiated 3T3-L1 adipocytes were incubated in serum-free DMEM containing 0.2% fatty acid–free BSA. Primary hepatocytes were obtained from B6 mice. Mouse hepatocytes were harvested and cultured as previously described (44). Min6 cells were provided by Christian Widmann (Department of Physiology, Université de Lausanne, Lausanne). They were maintained as previously described (45) and incubated in DMEM supplemented with 15% FBS and 5 mM glucose overnight. The day after, cells were incubated in serum-free DMEM containing 0.1% fatty acid–free BSA for 6 hours. C2C12 myoblasts were obtained from ATCC and were cultured in low-glucose DMEM with 10% FBS in 5% CO_2 in an incubator set at 37°C. For myotube differentiation, C2C12 myoblasts were seeded in 6-cm plates. When the cells reached 95% confluency, the culture medium was switched to DMEM containing 2% horse serum. The medium was changed every 2 days until day 5 of differentiation. C2C12 myotubes were incubated in α-MEM overnight to induce starvation. Primary hepatocytes, Min6 cells, and C2C12 myotubes were stimulated with insulin (100 nM).

Proteins extraction, coimmunoprecipitation assays, and immunoblot analyses. For endogenous immunoprecipitation experiments between CDK4 and IRS2, mature 3T3-L1 adipocytes were lysed in a buffer containing 0.3% CHAPS, 40 mM Hepes, pH 7.5, 120 mM NaCl, 1 mM EDTA, 10 mM pyrophosphate, 10 mM glycerophosphate, and protease inhibitor cocktail. Lysates were precleared with protein A/G–agarose beads (Life Technologies) and 4 μg of control antibody (HA antibody) for 1 hour. After this step, anti-CDK4 antibodies or HA antibodies were added to the precleared lysates overnight to immunoprecipitate CDK4 or for the control immunoprecipitation, respectively. For endogenous immunoprecipitation experiments, mature 3T3-L1 adipocytes or eWAT from mice was lysed in a buffer containing 20 mM Tris, pH 7.5, 150 mM NaCl, 1 mM EDTA, 1% Triton X-100, 1 mM Na_3VO_4, 1 mM β-glycerophosphate, 50 mM NaF, and protease inhibitor cocktail. Whole protein extracts were precleared with protein A/G–agarose beads (Life Technologies) for 1 hour, and anti-CDK4 antibody (Santa Cruz Biotechnology Inc., sc-260AC) and negative control (Rabbit IgG) (Santa Cruz Biotechnology Inc., sc-2345) were added to immunoprecipitate CDK4 overnight at 4°C. For IRS2 and PIK3R1 immunoprecipitation experiments from mature adipocytes and mice, whole protein extracts were precleared with protein A/G–agarose beads (Life Technologies) and 4 μg of control antibody (HA antibody) for 1 hour. Then anti-IRS2 anti-PIK3R1 antibody and negative control (HA antibody) were added to the precleared lysates for immunoprecipitation overnight at 4°C. Immunoprecipitation experiments in 293T cells were performed using the same buffer as above. Anti-CDK4 antibody (Santa Cruz Biotechnology Inc., sc-601) and negative control (rabbit IgG) (Santa Cruz Biotechnology Inc., sc-2027) were used for the immunoprecipitation. Flag-PIK3R1, Flag-PDKP1, and Flag-IRS2 were transfected with Lipofectamine 2000 (Invitrogen) and immunoprecipitated with Flag beads (Sigma-Aldrich A2220). Proteins were extracted with

the same lysis buffer described above and subjected to SDS-PAGE electrophoresis. Protein extractions from the different tissues (eWAT, BAT, brain, muscle, heart, kidney, lung, spleen, and liver) were prepared using M-PER mammalian extraction buffer (Thermo Scientific) containing 1:100 Halt phosphatase inhibitor cocktail (Thermo Scientific) and 1:100 Halt protease inhibitor cocktail, EDTA-free (Thermo Scientific). All the tissues were snap-frozen and then ground with Liquid N2 before lysis. The following antibodies were used for Western blot analysis: anti-CCND1 (NeoMarkers Rb-010-P0), anti-CCND3 (clone sc-6283), anti-CDK4 (clone sc-260), anti-HSL (clone sc-25843), anti-HA (clone sc-805), anti-IRS2 (clone sc-8299) (Santa Cruz Biotechnology Inc.); anti-CCND2 (clone ab3085), anti-CDK4 (clone DSC-35), anti-Ki67 (clone ab15580) (Abcam); anti–LMNA (clone 2032), anti–pHSL Ser573 (clone 4139), anti–RB1 Ser780 (clone 9307), anti–pAKT Thr308 (clone 4056), anti–pAKT Ser473 (clone 4060), anti-AKT (clone 9272), anti-CDK6 (clone DCS83) (Cell Signaling Technology); anti-Flag (clone F3165), anti-actin (clone A2066), anti-tubulin (clone T6199) (Sigma-Aldrich); anti-PI3K3R1 (clone 06-195) (Upstate); and anti-IRS2 (Millipore MABS15). The phosphospecific antibody against IRS2 Ser388 was synthesized and purchased from GenScript.

Kinase assays. 3T3-L1 mature adipocytes were incubated overnight in serum-free DMEM containing 0.2% fatty acid–free BSA and either stimulated with insulin (100 nM) or left untreated with lysates of these cells used to immunoprecipitate CDK4, as described above. Additionally, CDK4 was immunoprecipitated from eWAT collected from mice that had fasted for 16 hours and were injected i.p. with insulin (0.75 U/kg) for 30 minutes. Kinase assays were performed using immunoprecipitated CDK4 and 500 ng of recombinant RB1 protein (Santa Cruz Biotechnology Inc.) as a substrate in kinase buffer (25 mM Tris/HCl, pH 7.5, 150 mM NaCl, 10 mM $MgCl_2$, 1 mM DTT, 5 mM $Na_4P_2O_7$, 50 mM NaF, 1 mM vanadate, and protease inhibitor cocktail) with 40 μM ATP and 8 μCi γ-^{33P}ATP for 30 minutes at 30°C. Recombinant CDK4/CCND3 (ProQinase) was used as positive control. Boiling the samples for 5 minutes in the presence of denaturing sample buffer stopped the reaction. Samples were subsequently subjected to SDS-PAGE, and the gels were then dried in a gel dryer for 1 hour and exposed to an x-ray film at –80°C.

When using GST-purified proteins as substrates, kinase assays were performed using 500 ng of recombinant RB1 protein (Santa Cruz Biotechnology Inc.) as a positive control and recombinant CDK4/CCND3 kinase (ProQinase) and incubated in kinase buffer (described above) supplemented with 40 μM ATP and 8 μCi γ-^{33P}ATP for 30 minutes at 30°C.

PamChip peptide microarrays for kinome analysis following insulin stimulation. For kinome analysis, STK microarrays were purchased from PamGene International BV. Each array contained 140 phosphorylatable peptides as well as 4 control peptides. Sample incubation, detection, and analysis were performed in a PamStation 12 according to the manufacturer's instructions. Briefly, extracts from *Cdk4*$^{+/+}$and *Cdk4*^{nc} mice or mature 3T3-L1 adipocytes were made using M-PER mammalian extraction buffer (Thermo Scientific) containing 1:50 Halt phosphatase inhibitor cocktail (Thermo Scientific) and 1:50 Halt protease inhibitor cocktail, EDTA-free (Thermo Scientific), for 20 minutes on ice. The lysates were then centrifuged at 15,871 *g* for 20 minutes to remove all debris. The supernatant was aliquoted, snap-frozen in liquid nitrogen, and stored at –80°C until further processing. Prior to incubation with the kinase reaction mix, the arrays were blocked with 2% BSA in water for 30 cycles and washed 3 times with PK assay buffer. Kinase reactions were performed for 1 hour with 5 μg of total extract for the mouse experiment or 2.5 μg of total extract for the mature adipocyte and 400 μM ATP at 30°C. Phosphorylated peptides were detected with an anti-rabbit–FITC antibody that recognizes a pool of anti–phospho serine/threonine antibodies. The instrument contains a 12-bit CCD camera suitable for imaging of FITC-labeled arrays. The images obtained from the phosphorylated arrays were quantified using the BioNavigator software (PamGene International BV), and the list of peptides whose phosphorylation was significantly different between control (3 minutes of insulin treated in *Cdk4*$^{+/+}$ mice or 5 minutes of insulin stimulation in cells starved in the presence of DMSO) and test (3 minutes of insulin treated in *Cdk4*^{nc} mice or 5 minutes of insulin stimulation in cells starved in the presence of PD0332991) conditions was uploaded to GeneGo for pathway analysis. The list of the significantly different peptides is shown in Supplemental Figure 3, C and D. The BioNavigator software was used to perform the upstream STK analysis that is shown in Figure 4D.

Statistics. All statistics are described in the figure legends. The results were expressed as mean ± SEM. Pearson's correlation coefficient was calculated to test for correlation between 2 parameters. Comparisons between 2 groups were performed with an unpaired 2-tailed Student's *t* test, and multiple group comparisons were performed by 1-way ANOVA followed by Tukey's test and 2-way ANOVA followed by Tukey's test. $P < 0.05$ was considered significant.

Study approval. All animal care and treatment procedures were performed in accordance with Swiss guidelines and were approved by the Canton of Vaud SCAV (authorization VD 2627.b). For human samples, the protocol concerning the use of biopsy from patients was approved in agreement with Spanish regulations, either by the Ethics and Research Committee of Virgen de la Victoria Clinical University Hospital or by the Institutional Ethics Committee of the Joan XXIII University Hospital. All patients provided written informed consent.

Supplemental data. Additional methods information is available in Supplemental Experimental Procedures. The sequences of the primers used for RT-qPCR are available in Supplemental Table 1.

Author contributions

LF designed the project. SL, ICLM, PDD, XE, JCA, CC, AG, QL, LMC, BD, JSA, EB, SH, AA, and PD designed and carried out the experiments. The design and execution of the PamGene experiment was done by ICLM. VJ and FB provided the AAV8 vectors. LZ performed the tail-vein injections. CRK provided pBS mouse IRS-2 and *Irs2*$^{-/-}$ cells. JV and FJT provided human VAT samples. SL, ICLM, and LF wrote the manuscript.

Acknowledgments

Members of the Fajas laboratory are acknowledged for support and discussions. We thank M. Barbacid for providing *Cdk4*$^{R24C/R24C}$ and *Cdk4*^{nc} mice. We thank A.-C. Thomas and F. Thévenaz for technical support. We thank J.-C. Stehle from the Mouse Pathology Facility. F. Bosch is the recipient of an award from the ICREA Academia, Generalitat de Catalunya, Spain. Vector generation and production were funded by Ministerio de Economía y Competitividad (SAF 2014-54866-R), Spain. This work was supported by grants from the Swiss Ligue Contre le Cancer, the French Ligue

Contre le Cancer, and the Swiss National Science Foundation. S. Lagarrigue was supported by a grant from the French Ligue Contre le Cancer and the Swiss National Science Foundation.

Address correspondence to: Lluis Fajas, Department of Physiologie, Université de Lausanne, CH-1005 Lausanne, Switzerland. Phone: 41.21.692.55.10; E-mail: lluis.fajas@unil.ch.

1. Saltiel AR, Kahn CR. Insulin signalling and the regulation of glucose and lipid metabolism. *Nature*. 2001;414(6865):799–806.
2. White MF. Insulin signaling in health and disease. *Science*. 2003;302(5651):1710–1711.
3. Rask-Madsen C, Kahn CR. Tissue-specific insulin signaling, metabolic syndrome, and cardiovascular disease. *Arterioscler Thromb Vasc Biol*. 2012;32(9):2052–2059.
4. Vazquez-Vela ME, Torres N, Tovar AR. White adipose tissue as endocrine organ and its role in obesity. *Arch Med Res*. 2008;39(8):715–728.
5. Koch L, et al. Central insulin action regulates peripheral glucose and fat metabolism in mice. *J Clin Invest*. 2008;118(6):2132–2147.
6. Abel ED, et al. Adipose-selective targeting of the GLUT4 gene impairs insulin action in muscle and liver. *Nature*. 2001;409(6821):729–733.
7. Choi YH, et al. Alterations in regulation of energy homeostasis in cyclic nucleotide phosphodiesterase 3B-null mice. *J Clin Invest*. 2006;116(12):3240–3251.
8. Matsushime H, et al. Identification and properties of an atypical catalytic subunit (p34PSK-J3/cdk4) for mammalian D type G1 cyclins. *Cell*. 1992;71(2):323–334.
9. Connell-Crowley L, Harper JW, Goodrich DW. Cyclin D1/Cdk4 regulates retinoblastoma protein-mediated cell cycle arrest by site-specific phosphorylation. *Mol Biol Cell*. 1997;8(2):287–301.
10. Malumbres M, Barbacid M. Cell cycle, CDKs and cancer: a changing paradigm. *Nat Rev Cancer*. 2009;9(3):153–166.
11. Rane SG, et al. Loss of Cdk4 expression causes insulin-deficient diabetes and Cdk4 activation results in beta-islet cell hyperplasia. *Nat Genet*. 1999;22(1):44–52.
12. Serrano M, Hannon GJ, Beach D. A new regulatory motif in cell-cycle control causing specific inhibition of cyclin D/CDK4. *Nature*. 1993;366(6456):704–707.
13. Martin J, et al. Genetic rescue of Cdk4 null mice restores pancreatic beta-cell proliferation but not homeostatic cell number. *Oncogene*. 2003;22(34):5261–5269.
14. Abella A, et al. Cdk4 promotes adipogenesis through PPARgamma activation. *Cell Metab*. 2005;2(4):239–249.
15. Sarruf DA, Iankova I, Abella A, Assou S, Miard S, Fajas L. Cyclin D3 promotes adipogenesis through activation of peroxisome proliferator-activated receptor gamma. *Mol Cell Biol*. 2005;25(22):9985–9995.
16. Fajas L, Landsberg RL, Huss-Garcia Y, Sardet C, Lees JA, Auwerx J. E2Fs regulate adipocyte differentiation. *Dev Cell*. 2002;3(1):39–49.
17. Jimenez V, et al. In vivo adeno-associated viral vector-mediated genetic engineering of white and brown adipose tissue in adult mice. *Diabetes*. 2013;62(12):4012–4022.
18. Sotillo R, et al. Wide spectrum of tumors in knock-in mice carrying a Cdk4 protein insensitive to INK4 inhibitors. *EMBO J*. 2001;20(23):6637–6647.
19. Rodriguez-Diez E, et al. Cdk4 and Cdk6 cooperate in counteracting the INK4 family of inhibitors during murine leukemogenesis. *Blood*. 2014;124(15):2380–2390.
20. Zechner R, et al. FAT SIGNALS — lipases and lipolysis in lipid metabolism and signaling. *Cell Metab*. 2012;15(3):279–291.
21. Fry DW, et al. Specific inhibition of cyclin-dependent kinase 4/6 by PD 0332991 and associated antitumor activity in human tumor xenografts. *Mol Cancer Ther*. 2004;3(11):1427–1438.
22. Hilhorst R, Houkes L, Mommersteeg M, Musch J, van den Berg A, Ruijtenbeek R. Peptide microarrays for profiling of serine/threonine kinase activity of recombinant kinases and lysates of cells and tissue samples. *Methods Mol Biol*. 2013;977:259–271.
23. Thirone AC, Huang C, Klip A. Tissue-specific roles of IRS proteins in insulin signaling and glucose transport. *Trends Endocrinol Metab*. 2006;17(2):72–78.
24. Malumbres M, Barbacid M. Mammalian cyclin-dependent kinases. *Trends Biochem Sci*. 2005;30(11):630–641.
25. Viglietto G, et al. Cytoplasmic relocalization and inhibition of the cyclin-dependent kinase inhibitor p27(Kip1) by PKB/Akt-mediated phosphorylation in breast cancer. *Nat Med*. 2002;8(10):1136–1144.
26. Zhou BP, Liao Y, Xia W, Spohn B, Lee MH, Hung MC. Cytoplasmic localization of p21Cip1/WAF1 by Akt-induced phosphorylation in HER-2/neu-overexpressing cells. *Nat Cell Biol*. 2001;3(3):245–252.
27. Reuveni H, et al. Therapeutic destruction of insulin receptor substrates for cancer treatment. *Cancer Res*. 2013;73(14):4383–4394.
28. Baserga R. The insulin receptor substrate-1: a biomarker for cancer? *Exp Cell Res*. 2009;315(5):727–732.
29. Heilmann AM, et al. CDK4/6 and IGF1 receptor inhibitors synergize to suppress the growth of p16INK4A-deficient pancreatic cancers. *Cancer Res*. 2014;74(14):3947–3958.
30. Vora SR, et al. CDK 4/6 inhibitors sensitize PIK3CA mutant breast cancer to PI3K inhibitors. *Cancer Cell*. 2014;26(1):136–149.
31. Muranen T, Meric-Bernstam F, Mills GB. Promising rationally derived combination therapy with PI3K and CDK4/6 inhibitors. *Cancer Cell*. 2014;26(1):7–9.
32. Miller ML, et al. Drug synergy screen and network modeling in dedifferentiated liposarcoma identifies CDK4 and IGF1R as synergistic drug targets. *Sci Signal*. 2013;6(294):ra85.
33. Turner NC, et al. Palbociclib in hormone-receptor-positive advanced breast cancer. *N Engl J Med*. 2015;373(3):209–219.
34. Asghar U, Witkiewicz AK, Turner NC, Knudsen ES. The history and future of targeting cyclin-dependent kinases in cancer therapy. *Nat Rev Drug Discov*. 2015;14(2):130–146.
35. Menendez JA, Lupu R. Fatty acid synthase and the lipogenic phenotype in cancer pathogenesis. *Nat Rev Cancer*. 2007;7(10):763–777.
36. Copps KD, White MF. Regulation of insulin sensitivity by serine/threonine phosphorylation of insulin receptor substrate proteins IRS1 and IRS2. *Diabetologia*. 2012;55(10):2565–2582.
37. Solinas G, Naugler W, Galimi F, Lee MS, Karin M. Saturated fatty acids inhibit induction of insulin gene transcription by JNK-mediated phosphorylation of insulin-receptor substrates. *Proc Natl Acad Sci U S A*. 2006;103(44):16454–16459.
38. Sharfi H, Eldar-Finkelman H. Sequential phosphorylation of insulin receptor substrate-2 by glycogen synthase kinase-3 and c-Jun NH2-terminal kinase plays a role in hepatic insulin signaling. *Am J Physiol Endocrinol Metab*. 2008;294(2):E307–E315.
39. Fritsche L, et al. Insulin-induced serine phosphorylation of IRS-2 via ERK1/2 and mTOR: studies on the function of Ser675 and Ser907. *Am J Physiol Endocrinol Metab*. 2011;300(5):E824–E836.
40. Boura-Halfon S, Zick Y. Phosphorylation of IRS proteins, insulin action, and insulin resistance. *Am J Physiol Endocrinol Metab*. 2009;296(4):E581–E591.
41. Blanchet E, et al. E2F transcription factor-1 regulates oxidative metabolism. *Nat Cell Biol*. 2011;13(9):1146–1152.
42. Withers DJ, et al. Disruption of IRS-2 causes type 2 diabetes in mice. *Nature*. 1998;391(6670):900–904.
43. Tsuruzoe K, Emkey R, Kriauciunas KM, Ueki K, Kahn CR. Insulin receptor substrate 3 (IRS-3) and IRS-4 impair IRS-1- and IRS-2-mediated signaling. *Mol Cell Biol*. 2001;21(1):26–38.
44. Benhamed F, et al. The lipogenic transcription factor ChREBP dissociates hepatic steatosis from insulin resistance in mice and humans. *J Clin Invest*. 2012;122(6):2176–2194.
45. Annicotte JS, et al. The CDK4-pRB-E2F1 pathway controls insulin secretion. *Nat Cell Biol*. 2009;11(8):1017–1023.
